# Integrating Multifunctionalities into a 3D Covalent Organic Framework for Efficient CO_2_ Photoreduction

**DOI:** 10.1002/anie.202504772

**Published:** 2025-05-02

**Authors:** Ke Cheng, Shuo Kong, Jungeng Wang, Qiurong Wang, Shiling Yuan, Pei‐Zhou Li, Yanli Zhao

**Affiliations:** ^1^ School of Chemistry and Chemical Engineering, Shandong Provincial Key Laboratory for Science of Material Creation and Energy Conversion, Science Center for Material Creation and Energy Conversion Shandong University No. 27 Shanda South Road Ji'nan 250100 P.R. China; ^2^ School of Chemistry, Chemical Engineering and Biotechnology Nanyang Technological University 21 Nanyang Link Singapore 637371 Singapore

**Keywords:** Covalent organic frameworks, Organic cage, Photocatalytic CO_2_ reduction, Porphyrin, Single atom

## Abstract

Fabrication of highly efficient photocatalysts for CO_2_ conversion is still challenging. Herein, integrating nitrogen‐rich organic cages and the photoactive porphyrin moieties together, a 3D covalent organic framework (COF), Cage‐PorCOF, is successfully synthesized. After incorporating metal ions (Co^2+^ and Ni^2+^) into the cage‐based COF, Cage‐PorCOF(Co) and Cage‐PorCOF(Ni) are subsequently constructed for the CO_2_ photoreduction. Catalytic experiments show impressive performance in CO_2_ photoreduction with CO generation rates of up to 48 748 and 28 446 µmol g^−1^ h^−1^ in the first initiating hour for Cage‐PorCOF(Co) and Cage‐PorCOF(Ni), respectively, which is attributed to the synergistic effects from CO_2_‐affinity of the porous frameworks and incorporated metal atoms, the light‐absorption and charge separation ability of metalloporphyrin groups as well as the fully exposed single‐atomic catalytic sites confirmed by both experimental and theoretical analyses. This study demonstrates that by the integration of multiple functionalities into 3D porous solids, highly effective photocatalysts for CO_2_ conversion can be achieved.

## Graphical abstract

By integrating nitrogen‐rich supramolecular cages and porphyrin moieties as well as single‐atomic metal ions (Co^2+^ and Ni^2+^) together, two metalated photoactive covalent organic frameworks are constructed for efficient photocatalytic CO_2_ reduction.

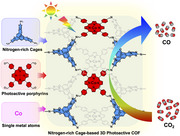

## Introduction

Carbon dioxide (CO_2_) neutralization technologies are urgently required to mitigate the effects of both the existing elevated atmospheric CO_2_ concentration and the continuous rise in CO_2_ emissions.^[^ ^1^, ^2^, ^3^, ^4^ ^]^ To this end, various strategies have been developed so far for the conversion of CO_2_ into value‐added products.^[^ ^5^, ^6^, ^7^ ^]^ CO_2_ conversion by photocatalytic approach stands out as an environmental‐friendly green way owing to the cleanliness, direct usability, and nonexhaustion of solar light as the usual energy source.^[^ ^8^, ^9^, ^10^, ^11^, ^12^ ^]^ Therefore, fabrication of efficient and selective photocatalysts for CO_2_ conversion becomes significantly important. Nevertheless, fundamental challenges still lie in the complicated process of photocatalytic CO_2_ conversion such as the limitations of a photocatalyst in the efficiency of CO_2_ phase transfer, light absorption and photocharge transfer, and the sufficient exposure of catalytic active centers.^[^ ^13^, ^14^, ^15^ ^]^ Although great efforts have been dedicated and research progresses have been made in the development of diverse photocatalysts,^[^ ^16^, ^17^, ^18^, ^19^ ^]^ large potentials in the photocatalytic efficiency and product selectivity remain difficult to be released due to the hardship to integrate all the benefits of CO_2_‐affinity functionalities, light absorption, and photocharge transfer moieties as well as fully‐exposed catalytic‐active sites into one system.

**Scheme 1:**
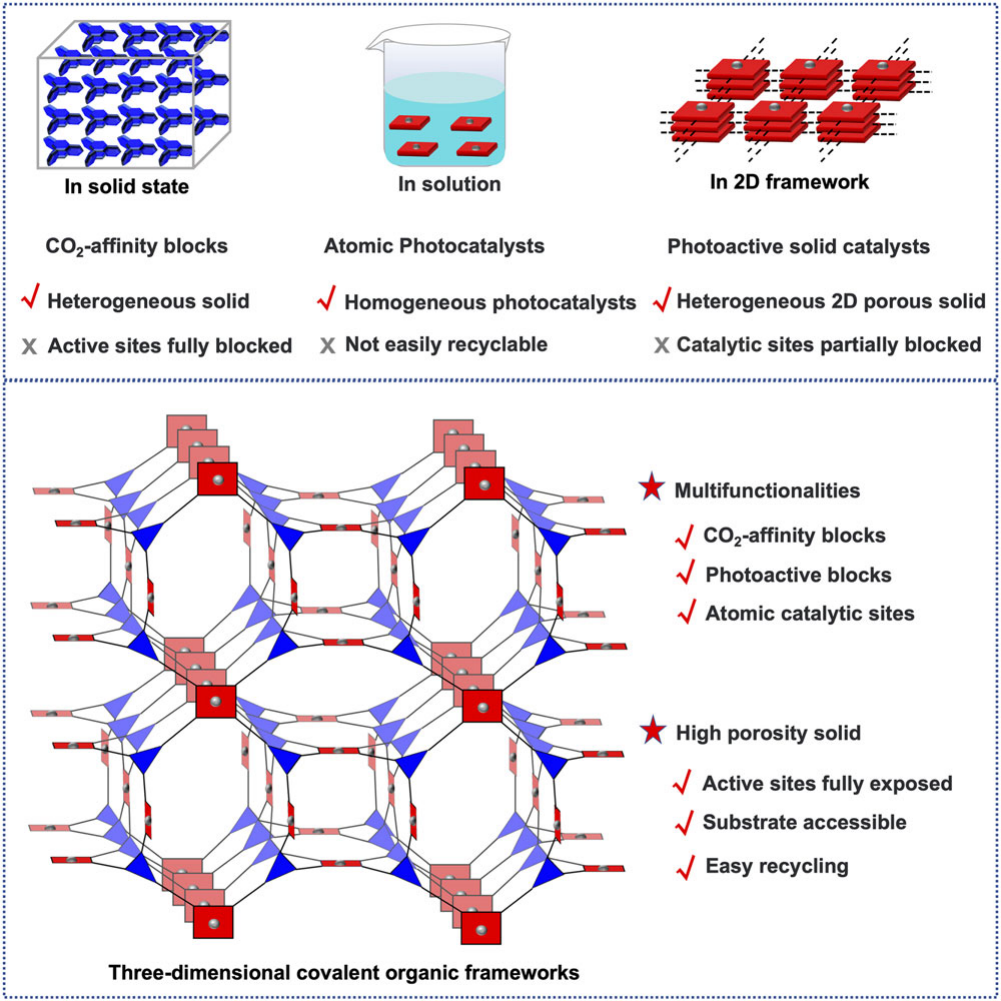
Illustration for the advantages of highly porous 3D covalent organic frameworks.

Covalent organic frameworks (COFs) have been emerging as a category of crystalline porous materials that are interconnected by multifunctional organic building blocks through covalent bonds.^[^ ^20^, ^21^, ^22^, ^23^, ^24^, ^25^ ^]^ The well‐defined crystalline porous structures together with tailored functionalities have endowed the COF materials superior potentials in diverse applications including heterogeneous catalysis.^[^ ^26^, ^27^, ^28^, ^29^, ^30^ ^]^ The unique feature of achieving complete control over both structure and functionality through meticulous selection of building blocks prior to synthesis makes the COFs highly attractive in the design of effective heterogeneous catalysts by integrating multifunctional moieties into one skeleton.^[^ ^31^, ^32^, ^33^ ^]^ Especially, comparing the densely packed solids, not‐easily recycled homogeneous solutions, and even vertical stacking 2D COFs, 3D COFs bare heterogeneous solid state with enhanced specific surface area, interconnected porous channels, and fully‐exposed functional moieties, which not only promote the diffusion of substrates and the sufficient accessibility of active sites but also well reserve the recyclability of heterogeneous catalysis (Scheme 1). Consequently, they have become promising platforms in design and construction of heterogeneous catalysts.^[^ ^34^, ^35^, ^36^, ^37^ ^]^ Therefore, the development of highly porous 3D COFs by integrating multifunctional moieties holds a high potential to fabricate photocatalysts for CO_2_ conversion.

Among the reported methods for construction of framework materials, the connection of the triple‐symmetrical and planar quadruple‐symmetrical organic models usually can give the formation of highly porous 3D frameworks (Scheme 1).^[^ ^38^, ^39^ ^]^ Therefore, selection of the triple‐symmetrical and planar quadruple‐symmetrical functional organic models to construct COFs should be a reliable way for fabrication of 3D COF‐based photocatalysts for CO_2_ conversion. Herein, a presynthesized triple‐symmetrical nitrogen‐rich organic cage as CO_2_‐affinity functionalities and the planar quadruple‐symmetrical porphyrin component as effective light absorption and photocharge transfer moiety are selected as the reactant organic building block,^[^ ^40^, ^41^, ^42^ ^]^ and a cage‐based porphyrin COF, denoted as Cage‐PorCOF, is successfully constructed after Schiff‐base condensation reaction. In order to enhance the catalytic performance in CO_2_ conversion, catalytic active metal ions of Co^2+^ and Ni^2+^ are then introduced into the host framework of the constructed Cage‐PorCOF by the postmetalation processes,^[^ ^43^ ^]^ and thereby two metalated COFs, Cage‐PorCOF(Co) and Cage‐PorCOF(Ni), are achieved for photocatalytic CO_2_ conversion.

Structural characterizations confirm the successful syntheses of the cage‐based highly porous 3D frameworks. Photophysical analyses reveal that they are potential semiconductors for photocatalytic CO_2_ reduction. Photocatalytic experiments show that all of them express high photocatalytic performance for CO_2_ reduction. Specifically, the Co coordinated Cage‐PorCOF(Co) achieves an impressive CO generation rate of up to 48 748 µmol g^−1^ h^−1^ with the selectivity of up to 97.9% in the first initiating hour using triethanolamine (TEOA) as the sacrificial agent in the presence of a photosensitizer. The catalyst can also be reused after a simple washing procedure for five cycles without remarkable decrease of the photocatalytic activity, demonstrating the excellent recyclability of the prepared heterogeneous photocatalyst. Comprehensive experiments and computational results elaborate on the synergistic effect of the nitrogen‐rich organic cages, photoresponsive porphyrin moieties, and single‐atomically dispersed metal sites, which can enhance the CO_2_ capture/activation capacity, light absorption, charge separation efficiency, and full utilization of catalytic sites for improving the activity of photocatalytic CO_2_ reduction. Thus, this work provides a reliable way to construct COF‐based effective photocatalysts for photocatalytic CO_2_ reduction.

## Results and Discussion

### Synthesis and Characterization of COFs

**Figure 1:**
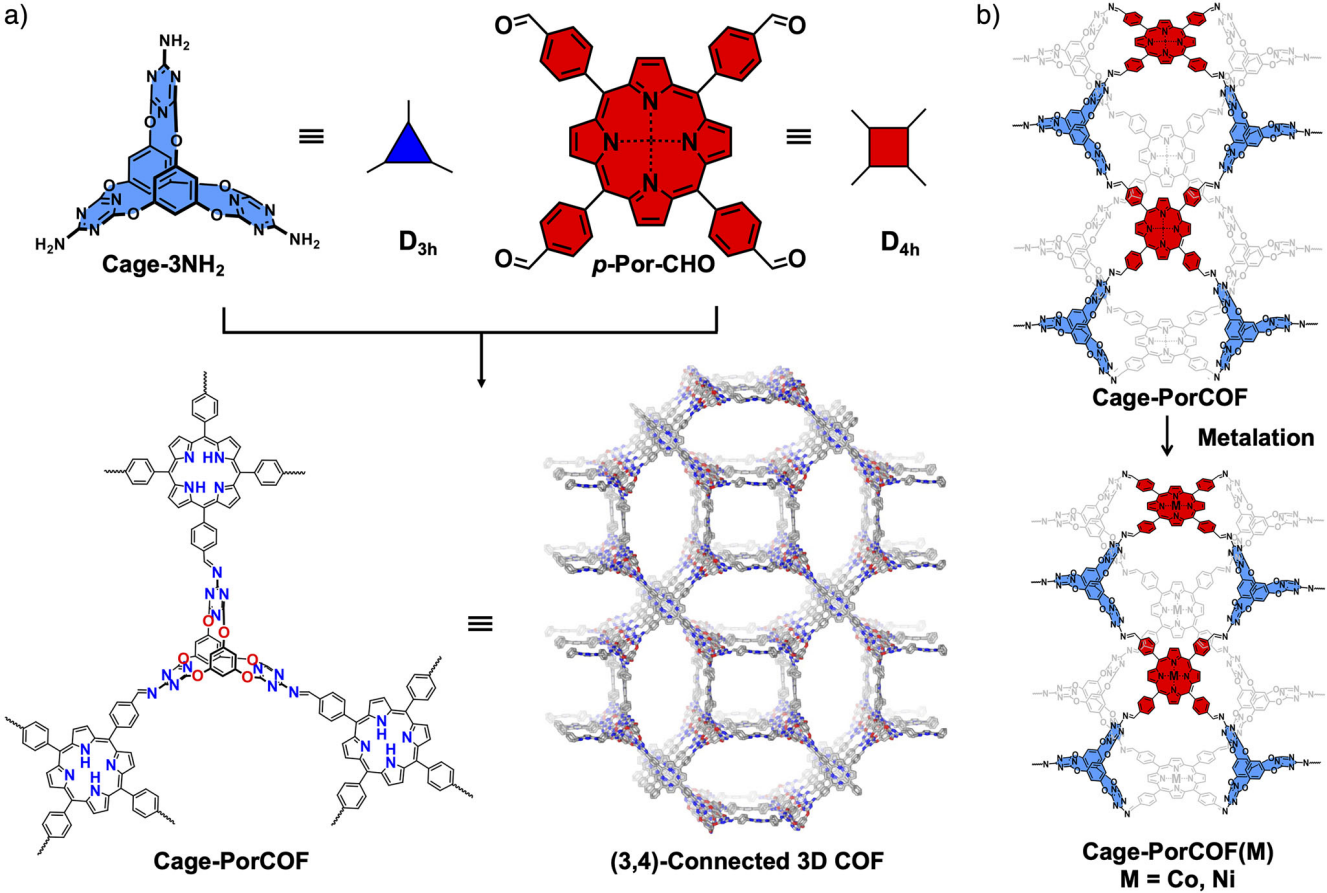
Schematic synthesis processes of a) Cage‐PorCOF and b) Cage‐PorCOF(Co) and Cage‐PorCOF(Ni).

The investigations of framework materials for CO_2_ related applications reveal that nitrogen‐rich moieties usually display high affinity toward CO_2_ molecule.^[^ ^44^ ^]^ In the nitrogen‐rich organic models, a group of organic cages possessing high‐density nitrogen‐rich core exhibit high potential affinity to CO_2_ through synergistic interactions of the hydrogen bonding with the two phloroglucinol aromatic protons and lone pair–π interactions enabled by the electron‐deficient triazines in the V‐clefts.^[^ ^45^, ^46^ ^]^ Therefore, a triple‐symmetrical nitrogen‐rich organic cage, i.e., Cage‐3NH_2_ (Figure 1a; Schemes S1 and S2), which we employed for COF constructions previously,^[^ ^47^, ^48^ ^]^ became the first preferred choice of us in constructing effective COF‐based photocatalysts to enhance the efficiency of CO_2_ phase transfer during the CO_2_ heterogeneous photocatalytic conversion process. In the planar quadruple‐symmetrical organic models, porphyrin derivative was the other choice due to its high absorption coefficient and exceptional energy and electron transfer capabilities, as well as the resultant prominent photophysical and photochemical properties.^[^ ^49^, ^50^ ^]^ Moreover, owing to their unique conjugated 18 π‐electron macrocyclic ring system, the porphyrin‐based compounds also demonstrate exceptional coordination abilities, allowing for the incorporation of various metal ions to act as catalytic active sites,^[^ ^51^ ^]^ which enable the obtained COFs exhibiting outstanding performance in photocatalysis.^[^ ^52^, ^53^, ^54^ ^]^ Therefore, the triple‐symmetrical nitrogen‐rich Cage‐3NH_2_ and the planar quadruple‐symmetrical 5,10,15,20‐tetrakis(4‐benzaldehyde) porphyrin (*p*‐Por‐CHO) were identified as reactants for the construction of highly porous photoactive COFs for photocatalytic CO_2_ conversion by Schiff‐base condensation reaction following the synthetic approach as shown in Figure 1 and Scheme S3. After the solvothermal reaction in a mixture solvent of dioxane (0.2 mL) and mesitylene (0.8 mL) with acetic acid as a catalyst at 120 °C for 5 days, Soxhlet extractions was carried out using tetrahydrofuran (THF) as the extraction agent for 24 h, followed by drying under vacuum at 60 °C overnight. Finally, purple solid of the nitrogen‐rich cage‐based porphyrin COF, Cage‐PorCOF, was prepared with a yield of around 70%.

The non‐noble metal ions of Co^2+^ or Ni^2+^ have been proven as effective catalytic active sites for photocatalytic CO_2_ reduction.^[^ ^52^, ^53^ ^]^ Therefore, Co^2+^ or Ni^2+^ was introduced into the synthesized Cage‐PorCOF through postsynthetic treatment with cobalt (II) acetate tetrahydrate or nickel (II) acetate tetrahydrate in ethanol, and the metalated crystalline frameworks, namely Cage‐PorCOF(Co) and Cage‐PorCOF(Ni), respectively, were achieved (Figure 1b). In a detail synthetic process,^[^ ^43^ ^]^ 15 mg of Cage‐PorCOF and 50 mg cobalt (II) acetate tetrahydrate or nickel (II) acetate tetrahydrate were dissolved in 20 mL ethanol. After the mixture being heated at 80 °C and refluxed under a N_2_ atmosphere for 12 h, the solutions were allowed to cool to room temperature and then the solid was filtered out followed by thoroughly washing with water and ethanol to remove the free metal ions. Finally, the solid of Cage‐PorCOF(Co) and Cage‐PorCOF(Ni) was obtained with the yields of 80%, and 85%, respectively, after being dried at 80 °C under dynamic vacuum overnight.

### Structures and Stability

**Figure 2:**
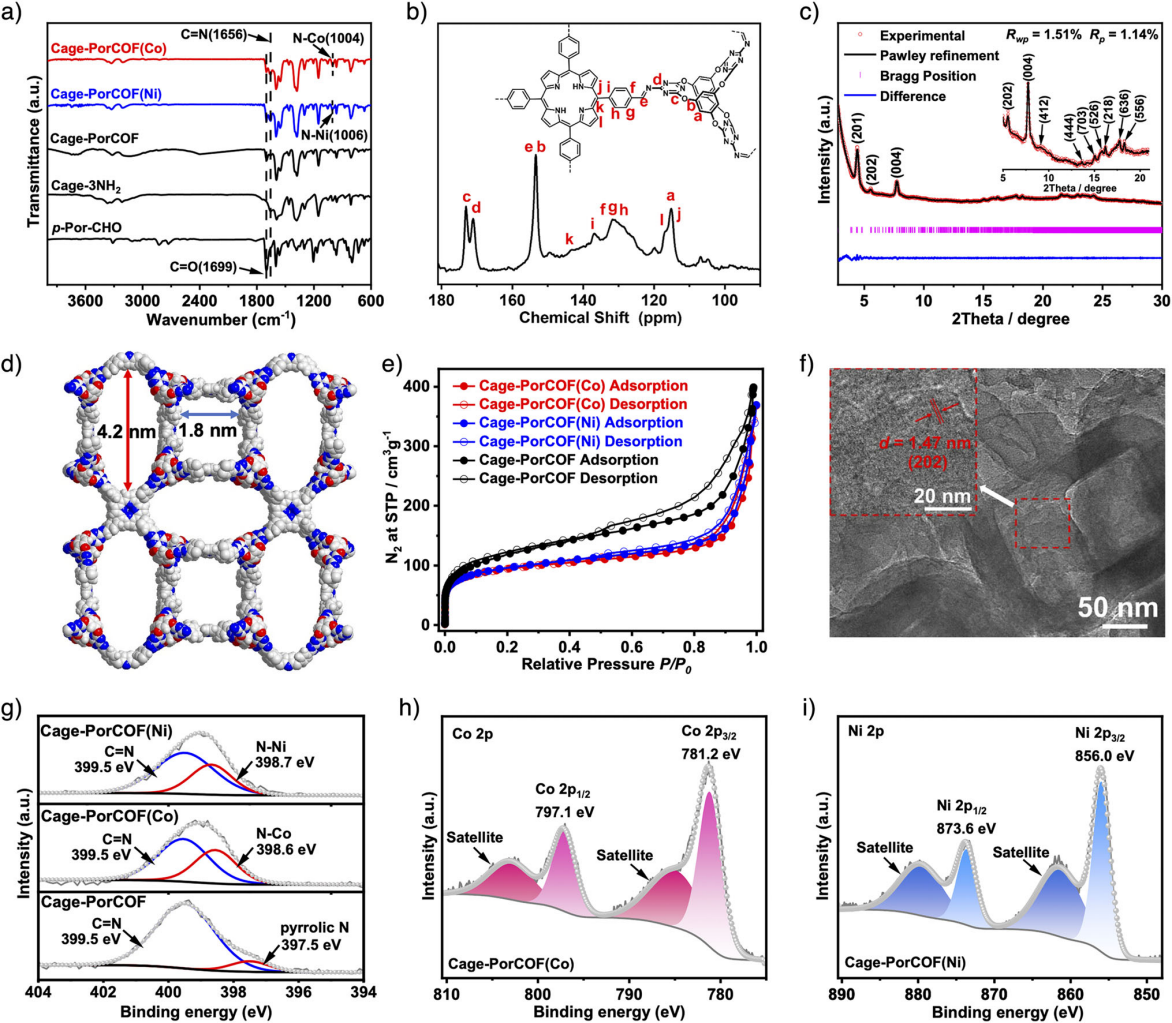
a) FT‐IR spectra of Cage‐PorCOF, Cage‐PorCOF(Co), Cage‐PorCOF(Ni), and the related reactants. b) Solid‐state ^13^C NMR spectrum of Cage‐PorCOF. c) PXRD patterns of Cage‐PorCOF with the experimental profiles in red, Pawley refined profiles in black, and the differences between the experimental and refined PXRD patterns in blue. d) Pore‐size visualization of Cage‐PorCOF. e) N_2_ sorption isotherms of the synthesized COFs at 77 K. f) TEM images of Cage‐PorCOF. g) Comparison of N 1s XPS spectra for the synthesized COFs. h) Co 2p XPS spectra of Cage‐PorCOF(Co). i) Ni 2p XPS spectra of Cage‐PorCOF(Ni).

In order to determine the formation of the synthesized COFs, Fourier transform infrared (FT‐IR) spectroscopy analyses were first employed on the synthesized Cage‐PorCOF and its modular precursors. As shown in Figure 2a, C═N stretching vibration peak in FT‐IR spectroscopy of Cage‐PorCOF was identified at 1656 cm^−1^, which was concomitant with the disappearance of the carbonyl band at 1699 cm^−1^ in the *p*‐Por‐CHO module, signifying the successful formation of the Schiff‐base imine bonds in the constructed Cage‐PorCOF after the condensation reaction of the Cage‐3NH_2_ and *p*‐Por‐CHO modules.^[^ ^47^, ^48^, ^55^ ^]^ In the solid‐state ^13^C cross‐polarization magic‐angle spinning (CP‐MAS) NMR spectra, prominent peaks at approximately 153 ppm (Figure 2b), corresponding to the characteristic carbon signals of the C═N linkages and the benzene ring bonded with oxygen in the organic cages, were also observed,^[^ ^56^ ^]^ which further confirmed the successful formation of Schiff‐base imine bonds in Cage‐PorCOF. In FT‐IR spectra, after incorporation of Co^2+^ or Ni^2+^ ions into the constructed Cage‐PorCOF, the appearance of new peaks at 1004 cm^−1^ for Cage‐PorCOF(Co) and 1006 cm^−1^ for Cage‐PorCOF(Ni) typically attributed to the coordination bonds between metal and polyazide centers in metal–porphyrin compounds,^[^ ^57^, ^58^ ^]^ indicating that the metal ions were successfully incorporated into the COF by coordination with porphyrin centers during the postsynthetic modification processes.

Subsequently, the crystalline structure of the synthesized Cage‐PorCOF was determined by powder X‐ray diffraction (PXRD) measurements with Cu Kα radiation in conjunction with structural simulations. As shown in Figure 2c, prominent peaks were clearly observed in the PXRD pattern of the Cage‐PorCOF, validating that the synthesized samples of the Cage‐PorCOF are in high crystallinity,^[^ ^12^ ^]^ further confirming that the 3‐connected cage‐based triple‐symmetrical amine and 4‐connected planar quadruple‐symmetrical aldehyde are connected through imine bonds to give the formation of a highly crystalline framework. The (3,4)‐connected structure models were then built by using the Materials Studio software package 2017.^[^ ^59^ ^]^ After comparing several possible interpenetrated and noninterpenetrated (3,4)‐connected nets^[^ ^60^, ^61^ ^]^ with different possible topologies (Figure S1), the detailed simulation clearly suggests that Cage‐PorCOF adopts a noninterpenetrated pto topology with a tetragonal *P‐*42*c* space group (Figures 1a and 2d). With the pto topological structure model, the intense peak at 4.40° and relatively weak peaks at 5.54° and 7.74° in the PXRD pattern of the Cage‐PorCOF were identified to the (201), (202), and (004) lattice planes, respectively (Figure 2c). Pawley refinement was then carried out, and the unit cell parameters of *a* = *b* = 44.61 Å, *c* = 45.62 Å, *α* = *β* = *γ* = 90° were obtained, which led to a full profile pattern matching with satisfactorily low residual values and acceptable profile differences (*R*_p_ = 1.14%, *R*_wp_ = 1.51%, Figure 2c and Table S1). The synthesized samples of Cage‐PorCOF(Co) and Cage‐PorCOF(Ni) exhibited similar PXRD patterns with the main peaks observed at 4.42°, 5.54°, and 7.80° for Cage‐PorCOF(Co) and 4.43°, 5.56°, and 7.76° for Cage‐PorCOF(Ni) (Figure S2), indicating the crystalline structures were well maintained after the postmetalation processes. Moreover, no diffraction peaks of Co/Ni nanoparticles or Co/Ni salt were observed, suggesting that Co/Ni were well coordinated with porphyrin centers rather than in the forms of metal nanoparticles in the metalated COFs.^[^ ^62^ ^]^

After confirming the thermal stability of up to approximately 430 °C by thermogravimetric analysis (TGA) (Figure S3) and chemical stability by immersing in various commonly used solvents followed by PXRD measurements (Figure S4) and FT‐IR (Figure S5) analyses, the synthesized COFs were activated by solvent immersion followed by degassing under vacuum at 120 °C for 24 h.^[^ ^63^ ^]^ Then, N_2_ sorption measurements at 77 K were conducted to evaluate the porosity of the constructed COFs. As shown in Figure 2e, the synthesized Cage‐PorCOF exhibited a high N_2_ uptake capability with rapid increases at lower relative pressure ranges (*P*/*P*_0_ < 0.1) followed by a plateau along with the increase of the relative pressure, indicating its microporous features. Hysteresis was also observed at higher relative pressures (*P*/*P*_0_ > 0.4), suggesting it possessed mesoporous pores.^[^ ^64^ ^]^ Based on the N_2_ sorption isothermal, the Brunauer–Emmett–Teller (BET) surface area of Cage‐PorCOF was determined to be 415 m^2^ g^−1^ with pore size distributions centered at about 1.8 and 4.2 nm (Figures 2d and S6). Interestingly, owing to the highly porous nature of pto frameworks,^[^ ^64^ ^]^ the simulated structure of Cage‐PorCOF also exhibited two types of pores, one in microporous size of 1.8 nm and the other in a mesoporous size with pore aperture of approximately 4.2 nm, which are consistent very well with the results calculated from the N_2_ sorption measurements. It further proved the correctness of the simulated structure model of Cage‐PorCOF. After loading Co^2+^ and Ni^2+^ ions into Cage‐PorCOF, the BET surface areas of the obtained Cage‐PorCOF(Co) and Cage‐PorCOF(Ni) slightly decreased to 316 and 324 m^2^ g^−1^, respectively. Similar pore size distributions as in the Cage‐Por‐COF were also observed in both samples of Cage‐PorCOF(Co) and Cage‐PorCOF(Ni) (Figures S7 and S8), further revealing that the frameworks were well maintained after incorporation of cobalt and nickel ions into Cage‐PorCOF.

The morphologies of the synthesized COFs were then directly examined by the scanning electron microscopy (SEM) measurements. Aggregated regular structures with the size range from 500 nm to 1.5 µm were observed in the SEM images of Cage‐PorCOF (Figure S9). After being treated by ultrasonication in ethanol, the well‐dispersed dried COF samples on copper grids were also detected by high‐resolution transmission electron microscopy (TEM) measurements. The obtained TEM images clearly displayed the lattice fringes of Cage‐PorCOF, further confirming the long‐range ordered crystalline structure of the synthesized Cage‐PorCOF. The lattice spacing was estimated to be 1.47 nm, which corresponded to the (202) crystal plane (Figure 2f). The SEM and TEM images of Cage‐PorCOF(Co) and Cage‐PorCOF(Ni) exhibited a similar morphology without obvious changes to that of Cage‐PorCOF, further indicating that the crystalline structure was well maintained after the postmetalation processes (Figures S10–S13). Elemental mapping images determined by energy‐dispersive X‐ray spectroscopy (EDS) indicated that the elements of Co and Ni as well as other elements of C, N, and O were uniformly distributed in the samples of Cage‐PorCOF(Co) and Cage‐PorCOF(Ni) (Figures S10 and S11), respectively, which should be attributed to the coordination between metal ions with porphyrin centers. Inductively coupled plasma optical emission spectrometer (ICP‐OES) analyses determined 2.88 wt% Co content in Cage‐PorCOF(Co) and 3.05 wt% Ni content in Cage‐PorCOF(Ni), which were consistent well with the theoretical value of coordinated ions in the samples of Cage‐PorCOF(Co) and Cage‐PorCOF(Ni), indicating the Co/Ni were coordinated with porphyrin centers rather than in the forms of metal nanoparticles or metals in the metalated COFs.

X‐ray photoelectron spectroscopy (XPS) measurements were employed to investigate the chemical composition and electronic valence states of loaded metal ions in the samples of both Cage‐PorCOF(Co) and Cage‐PorCOF(Ni). Comparing with the full XPS spectrum of Cage‐PorCOF, additional peaks corresponding to Co or Ni elements appeared in the full XPS spectra of Cage‐PorCOF(Co) or Cage‐PorCOF(Ni), confirming the successful incorporation of Co or Ni ions into the COF frameworks (Figures S14–S16). As depicted in Figure 2g, the high‐resolution N 1s XPS spectra of Cage‐PorCOF were deconvoluted into two peaks at 399.5 and 397.5 eV, which were attributed to the C═N bonds and pyrrolic N in the porphyrin center, respectively. The disappearance of the pyrrolic N peak and the appearance of the N–Co peak in Cage‐PorCOF(Co) suggested the formation of coordination bonds between the porphyrin centers and the introduced cobalt ions.^[^ ^62^ ^]^ The same results were also achieved from the observation in the high‐resolution N 1s XPS spectrum of the Cage‐PorCOF(Ni) sample. Furthermore, the Co 2p and Ni 2p XPS spectra exhibited divalent oxidation states features along with their associated satellite character for both Co^2+^ and Ni^2+^ species (Figure 2h,i), indicating the loaded metal species were still in the oxidation states as those in the metal salt precursors.^[^ ^10^ ^]^

### Photochemical Property

**Figure 3:**
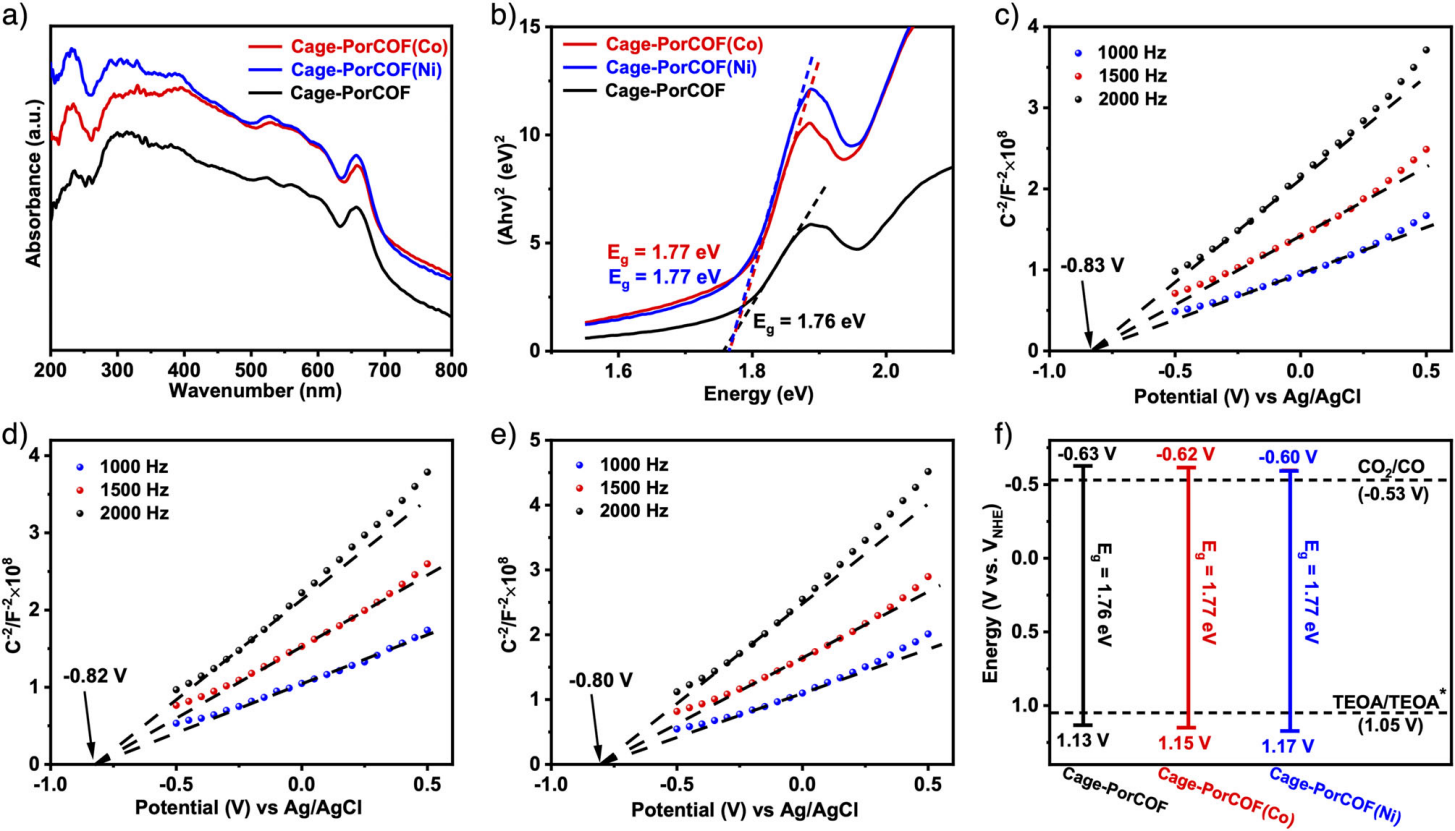
a) UV–vis DRS spectra and b) Tauc plots for the synthesized COFs. Mott–Schottky plots for c) Cage‐PorCOF, d) Cage‐PorCOF(Co), and e) Cage‐PorCOF(Ni). f) Schematic band structure diagram based on UV–vis DRS spectra and Mott–Schottky plots for the synthesized COFs.

For photocatalytic CO_2_ conversion, in principle, when the bandgap of a photocatalyst is lower than the redox potential of the CO_2_ reduction, it can be used as the catalyst for the photocatalytic CO_2_ reduction.^[^ ^10^ ^]^ Therefore, the photophysical properties and bandgaps of all obtained COFs were then investigated by UV–vis diffuse reflectance spectroscopy (DRS) and Mott–Schottky plots measurements. As shown in Figures 3a, S17, and S18, all of the obtained COFs exhibited a broad UV–visible light absorption band, which should be attributed to the selection of the porphyrin‐based *p*‐Por‐CHO as the planar quadruple‐symmetrical reactant. Moreover, the visible‐light absorption intensities of the Cage‐PorCOF(Co) and Cage‐PorCOF(Ni) were stronger than that of pristine Cage‐PorCOF, implying that the incorporation of metals contributed to their visible light absorption probably because of the increased degree of delocalization based on the chelation of metal species.^[^ ^65^ ^]^ The optical bandgaps of all obtained COFs calculated from the Tauc plot were found to be 1.76, 1.77, and 1.77 eV for Cage‐PorCOF, Cage‐PorCOF(Co), and Cage‐PorCOF(Ni), respectively (Figure 3b). Comparing with Cage‐PorCOF, the incorporation of metal ions improves the light‐harvesting properties of the Cage‐PorCOF(Co) and Cage‐PorCOF(Ni).^[^ ^66^ ^]^

Mott–Schottky plots of all obtained COFs displayed the positive slopes, typical of n‐type semiconductors,^[^ ^17^ ^]^ with flat band potentials at −0.83, −0.82, and −0.80 V [versus Ag/AgCl electrodes] for Cage‐PorCOF, Cage‐PorCOF(Co), and Cage‐PorCOF(Ni), respectively (Figure 3c–e). Their conduction bands were −0.63, −0.62, and −0.60 V, based on normal hydrogen electrode (NHE). Subsequently, their valence band values were calculated by combining with the optical bandgap values obtained from the Kubelka–Munk transformed reflectance spectra and Mott–Schottky tests. Finally, the schematic diagram of the energy band structure of all obtained COFs is depicted in Figure 3f. The conductive band potentials of these three Cage‐based COFs were lower than the redox potential of CO_2_ to CO (−0.53 V) but much higher than the lowest unoccupied molecular orbital energy level (−1.31 V versus NHE) of the photosensitizer [Ru(bpy)_3_]Cl_2_.^[^ ^10^ ^]^ Thus, the photoinduced electrons could transfer from the photosensitizer [Ru(bpy)_3_]Cl_2_ to COF, and the CO_2_RR is thermodynamically feasible, indicating they are semiconductor photocatalysts for photocatalytic CO_2_ reduction to CO.

### Photocatalytic CO_2_ Reduction

**Figure 4:**
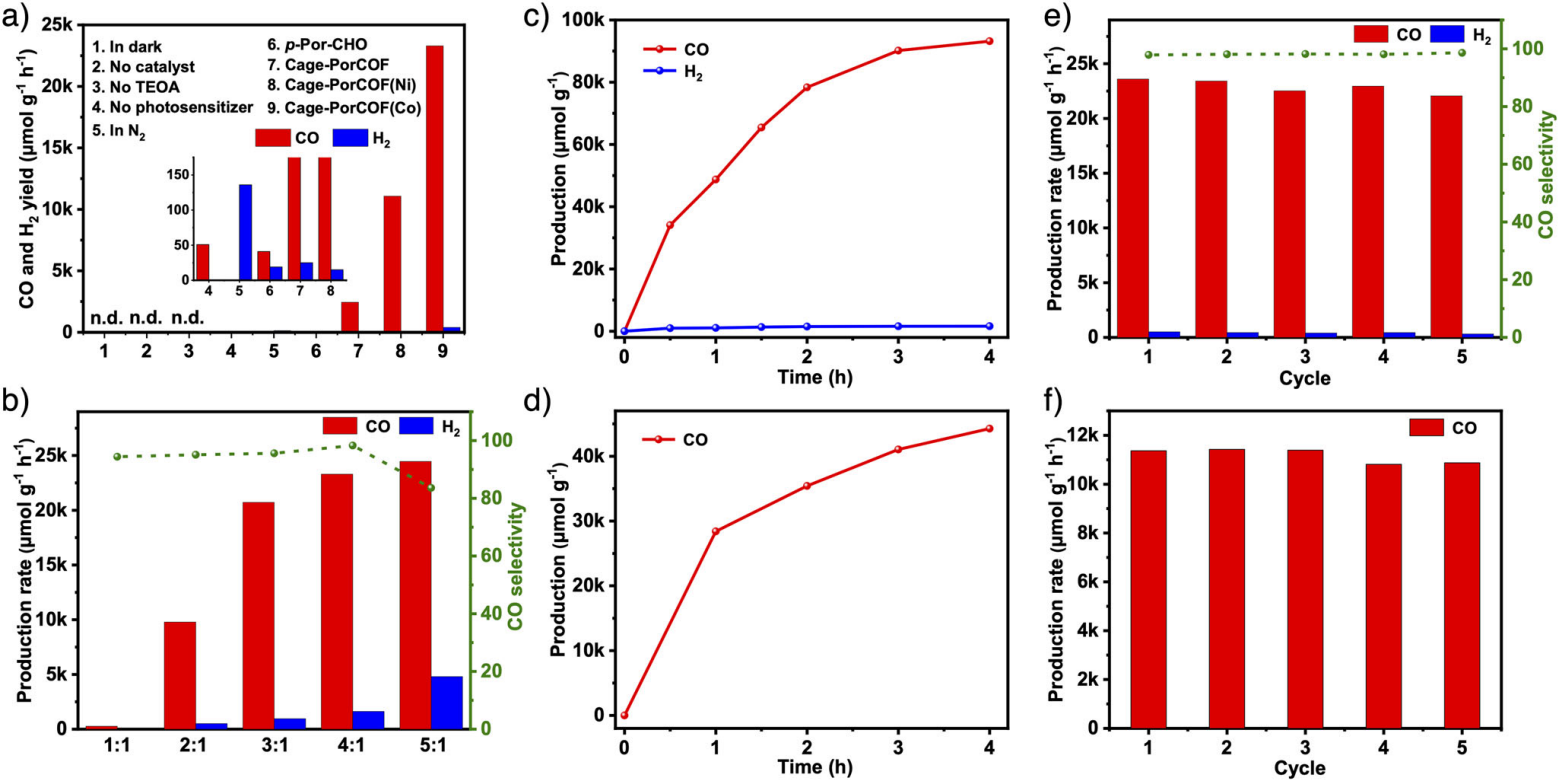
a) Control experiments of catalytic conditions. n.d., not detected. b) Effect of the acetonitrile/H_2_O volume ratio on the photocatalytic CO_2_RR and c) time‐dependent CO and H_2_ generation catalyzed by Cage‐PorCOF(Co). d) Time‐dependent CO generation catalyzed by Cage‐PorCOF(Ni). Durability measurements of e) Cage‐PorCOF(Co) and f) Cage‐PorCOF(Ni) for CO_2_ photoreduction.

Keeping all these positive results in hand, experiments of photocatalytic CO_2_ reduction were performed in a CO_2_‐saturated acetonitrile/water (MeCN/H_2_O) mixed solution under 300 W Xe lamp irradiation, with [Ru(bpy)_3_]Cl_2_ and TEOA as the potential photosensitizer and electron donor, respectively. Firstly, a series of control experiments were performed to optimize the photocatalytic reaction conditions. As shown in Figure 4a, it was found that no CO was produced without visible light, catalyst, or TEOA, and only a trace amount of CO was detected in the absence of a photosensitizer. A small amount of CO was detected when using *p*‐Por‐CHO and Cage‐PorCOF as catalysts, which should be attributed to the lack of catalytic metal sites. When Cage‐PorCOF(Co) or Cage‐PorCOF(Ni) and [Ru(bpy)_3_]Cl_2_ simultaneously existed in the system, the CO production was significantly increased. These results indicated that the photocatalysts of Cage‐PorCOF(Co) or Cage‐PorCOF(Ni), photosensitizers, and electron donors are all essential for photocatalytic CO_2_ reduction to CO. Considering the good solubility of MeCN for CO_2_ and its possible cation‐solvating property beneficial for the electron transfer to CO_2_,^[^ ^67^ ^]^ the effect of MeCN on photocatalytic activity and CO selectivity was also explored by adjusting its amount in the MeCN/H_2_O solution, and finally the optimized volume ratio of MeCN/H_2_O was determined to 4:1 (Figure 4b). Under the optimized conditions, photocatalytic CO_2_ reduction to CO was performed, and the gaseous and liquid products were detected by gas chromatography and ^1^H NMR spectroscopy to detect the gaseous and liquid products, respectively. H_2_ and CO were detected in the gaseous products, but no liquid product was detectable (Figures S19 and S20).

Under the optimal condition, photocatalytic reduction of CO_2_ to CO was further carried out. With Cage‐PorCOF(Co) as the photocatalyst, the product of CO with a slight of H_2_ was generated, and the yields of CO and H_2_ changed along with the reduction time. Under the light illumination, a sharp increasing CO yield as high as up to 48 748 µmol g^−1^ with a CO selectivity of 97.9% within the first initiating hour of the reaction was detected (Figure 4c). The CO production was still highly produced within 4 h continuous test, although the production rate was slightly decreased as the second hour comparing with the CO production rate of the first initiating hour. Based on the 4 h continuous test, Cage‐PorCOF(Co) can product CO of 93 180 µmol g^−1^ with an average CO generation rate of even up to 23 295 µmol g^−1^ h^−1^. Such an effective performance makes the synthesized Cage‐PorCOF(Co) one of the most effective COF‐based photocatalysts for photocatalytic CO_2_ reduction (Table S2).^[^ ^10^, ^12^, ^68^ ^]^ When changing the photocatalyst of Cage‐PorCOF(Co) to Cage‐PorCOF(Ni), a CO generation rate of up to 28 446 µmol g^−1^ at the first initiating hour and CO product of 44 324 µmol g^−1^ for 4 h continuous test in an average CO generation rate of 11 081 µmol g^−1^ h^−1^ were obtained (Figure 4d), the values of which also make Cage‐PorCOF(Ni) one of the most effective COF‐based photocatalysts for photocatalytic CO_2_ reduction (Table S2).^[^ ^69^, ^70^ ^]^ Besides CO as the major product, nearly no H_2_ and only trace CH_4_ were detected within the whole process of 4 h continuous test with Cage‐PorCOF(Ni) as the photocatalyst, which means that Cage‐PorCOF(Ni) can achieve nearly 100% selectivity in photocatalytic reduction CO_2_ to CO.

Taking ^13^CO_2_ as reactant, the produced ^13^CO (*m/z* = 29) was detected by gas chromatography–mass spectrometry, confirming that the generated CO came from CO_2_ rather than other organic species, such as the photosensitizer [Ru(bpy)_3_]Cl_2_, electron donor TEOA, or COF (Figures S21 and S22). Additionally, the apparent quantum efficiency (AQE) of the photocatalytic CO_2_ reduction process over Cage‐PorCOF(Co) was studied at various wavelengths (Figure S23) and the highest AQE value was of 2.67% at 450 nm.

The CO_2_ reduction stability of Cage‐PorCOF(Co) and Cage‐PorCOF(Ni) was evaluated by a cycling test. As shown in Figure 4e, no remarkable change was observed on the CO yield rate and selectivity with Cage‐PorCOF(Co) as the photocatalyst within the five rounds with each cycle in 4 h continuous test, proving its cycling durability and reusability for photocatalytic CO_2_ reduction. The same result was also observed in the cycling experiment with Cage‐PorCOF(Ni) as the photocatalyst (Figure 4f). After cycling experiments, characterizations were also carried out to detect the framework stability and the results revealed that comparing with the related fresh samples, nearly no change was observed from the SEM morphologies as well as FT‐IR spectra of the recollected samples (Figures S24–S26). Moreover, Co 2p XPS spectrum of recovered Cage‐PorCOF(Co) was also detected and also no change can be observed compared with that of the fresh sample (Figure S27), suggesting that the Co sites were strongly chelated in the porphyrin centers of the synthesized Cage‐PorCOF(Co). The same result was also detected for the recovered sample of Cage‐PorCOF(Ni) (Figure S28). Additionally, the PXRD measurements of the recollected Cage‐PorCOF(Co) and Cage‐PorCOF(Ni) showed that their crystallinity was still well maintained after the experiments of photocatalytic CO_2_ reduction (Figure S29). All of these characterizations clearly demonstrated the high framework stability of the synthesized Cage‐PorCOF(Co) and Cage‐PorCOF(Ni) in the photocatalytic conditions.

### Reaction Mechanism

**Figure 5:**
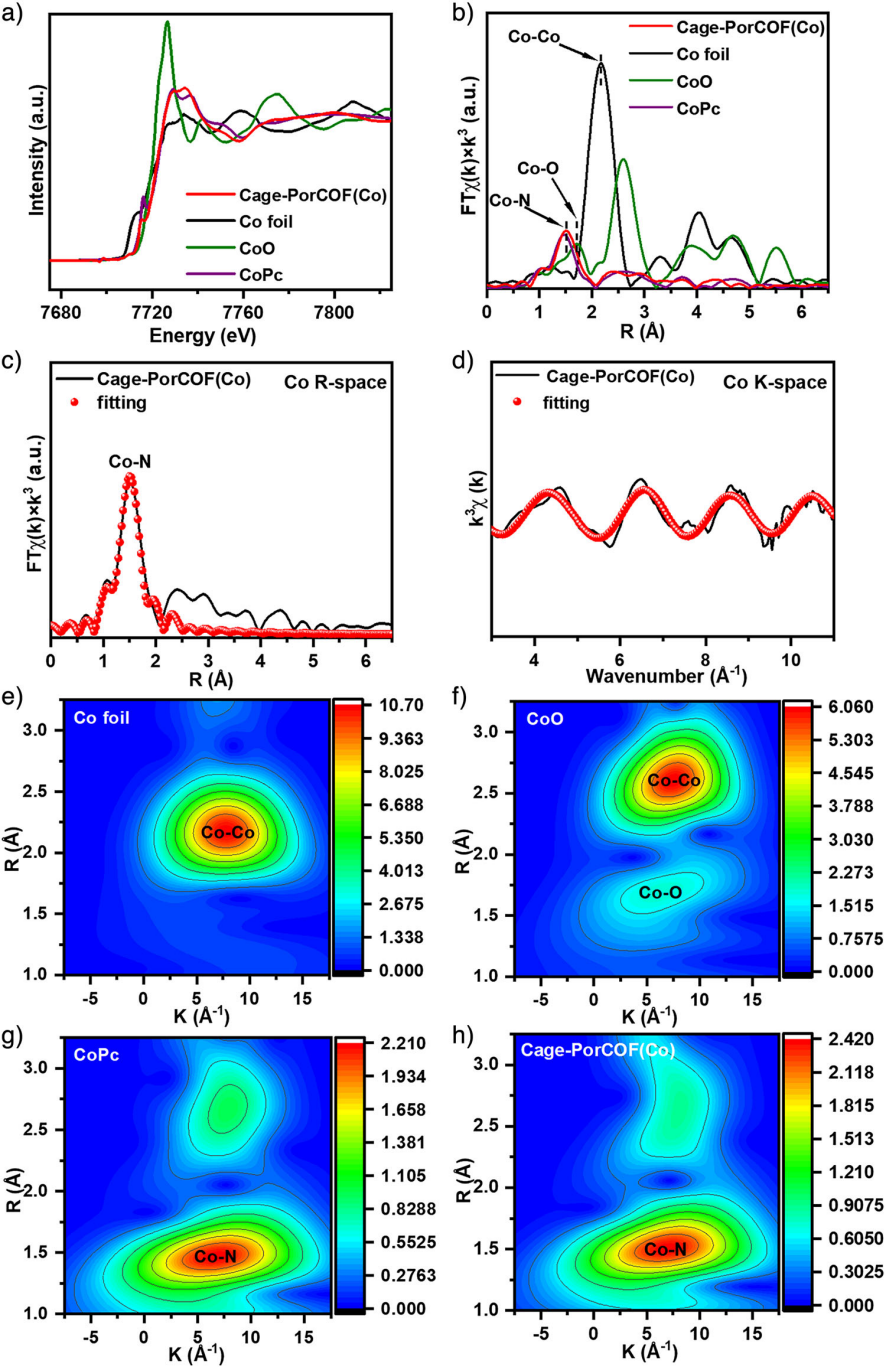
a) XANES and b) FT‐EXAFS spectra of Cage‐PorCOF(Co), Co Foil, CoO, and CoPc. EXAFS fitting results of Cage‐PorCOF(Co) at c) R‐space and d) K‐space. Wavelet transform of e) Co Foil, f) CoO, g) CoPc, and h) Cage‐PorCOF(Co).

**Figure 6:**
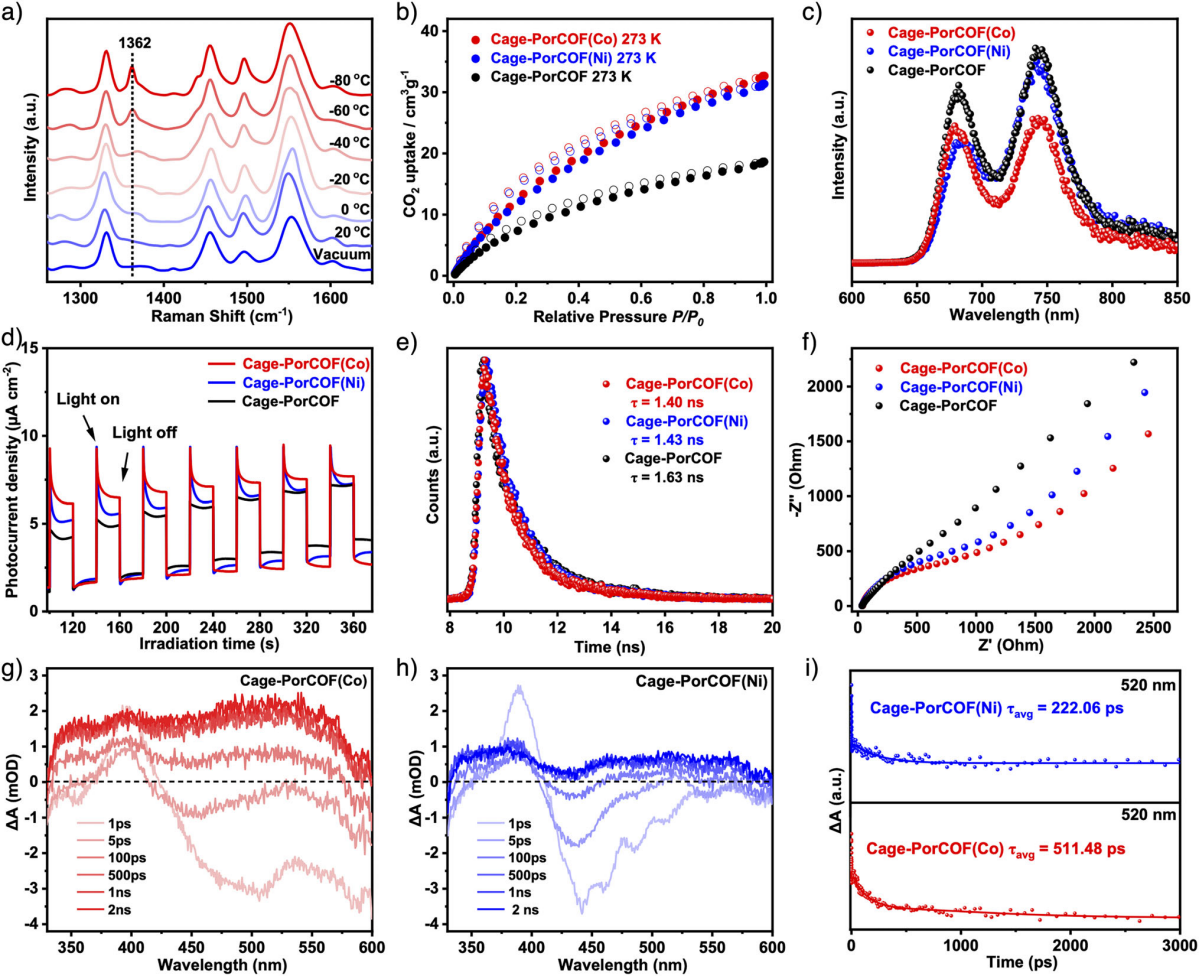
a) In situ Raman spectra of CO_2_ interaction with Cage‐PorCOF. b) CO_2_ sorption isotherms, c) steady‐state PL spectra, d) transient photocurrent responses, e) time‐resolved PL spectra, and f) EIS Nyquist plots of the synthesized COFs. Fs‐TAS of g) Cage‐PorCOF(Co) and h) Cage‐PorCOF(Ni) in the time range of 1 ps–2 ns. i) Fs‐TAS decay kinetic curves of Cage‐PorCOF(Co) and Cage‐PorCOF(Ni) at 520 nm.

The high photocatalytic CO_2_ reduction performance of Cage‐PorCOF(Co) inspired us to investigate its causation by various characterizations. As the active catalytic sites, the existence form of the Co atoms was conducted by synchrotron‐radiation‐based X‐ray absorption near‐edge spectroscopy (XANES) and extended X‐ray absorption fine structure (EXAFS) measurements. As shown in Figure 5a, the XANES spectra reveal that the Co K‐edge of Cage‐PorCOF(Co) lies between the K‐edges of CoO and CoPc, suggesting that the oxidation state of Co in this material is +2, which is consistent well with the aforementioned XPS analysis. The EXAFS data (Figure 5b) show the Fourier transform (FT) of the Co K‐edge, where a prominent peak at 1.50 Å is observed, corresponding to Co–N scattering path in CoPc, indicating that Co atoms in Cage‐PorCOF(Co) are coordinated by N atoms. No evidence of Co–Co bonding was detected, ruling out the presence of Co nanoparticles or clusters and confirming the single‐atom nature of Co catalytic center in Cage‐PorCOF(Co).^[^ ^10^ ^]^ The coordination number and local structure of the Co single atoms were determined by fitting the first shell in the EXAFS analysis. From the fitting of the K‐space data (Figures 5c,d and S30 as well as Table S3), it is clear that Co adopts a Co–N_4_ coordination configuration. Additionally, wavelet transform (WT) analysis provided further insight into the coordination environment of the metal species. As shown in Figure 5e–h, the maximum intensities for Co─Co, Co─O, and Co─N bonds in Co foil, CoO, and CoPc were observed at 7.75, 6.45, and 6.85 Å⁻¹, respectively. For Cage‐PorCOF(Co), the maximum intensity appeared at 6.95 Å^−1^, corresponding to the Co─N bond. These results provide strong evidence for the stable Co–N_4_ coordination mode of Co ions within the porphyrin units of Cage‐PorCOF(Co) rather than in the forms of metal nanoparticles, indicating that the Co catalytic centers are regularly distributed in a 3D framework in single‐atom form for a full exposure of each catalytic site in the catalytic process. As CO_2_ sorption was the first step among the whole process of photocatalytic CO_2_ reduction, the CO_2_ adsorption behavior of the COFs was then performed. To avoid interference from other factors such as metal ions, the synthesized metal‐free COF, Cage‐PorCOF, was tested by in situ Raman spectroscopy measurements at room and lower temperatures in a vacuum or 1 atm CO_2_ environment. As shown in Figure 6a, no sign of CO_2_ was detected under vacuum condition, whereas a band at 1362 cm^−1^ gradually becomes stronger with the decrease of the temperature. Based on the literature description,^[^ ^27^, ^44^ ^]^ the generated peak at 1362 cm^−1^ should be corresponding to the symmetric C═O stretch mode of the adsorbed CO_2_ in the framework, indicating that the synthesized Cage‐PorCOF has a significant affinity toward gaseous CO_2_ molecule and can promote phase transfer of CO_2_ molecule in the photocatalytic CO_2_ reduction.

To further investigate the influence of incorporated single‐metal atoms on the CO_2_ molecules, we tested the CO_2_ adsorption capabilities of all three COF samples at 273 and 298 K (Figures 6b and S31). Both of Cage‐PorCOF(Co) and Cage‐PorCOF(Ni) display remarkably higher CO_2_ uptake capabilities than that of the metal‐free Cage‐PorCOF at both 273 and 298 K, proving that the incorporation of metal ions can prominently promote the CO_2_ adsorption capabilities and there are stronger interactions of CO_2_ molecule with the incorporated atomic metal sites within the metalated Cage‐PorCOF(Co) and Cage‐PorCOF(Ni). The CO_2_ adsorption experiments clearly demonstrated that both Cage‐PorCOF itself and the incorporated metal ions were beneficial in transferring abundant gaseous CO_2_ molecule to participate in the following CO_2_ catalytic steps.^[^ ^27^ ^]^

It is known that upon illumination with visible light, the electrons in semiconducting materials could move to the conduction band to create holes in the valence band. The electron‐hole separation efficacy was one of the most important factors for CO_2_ reduction.^[^ ^71^ ^]^ Thus, the photogenerated electron‐hole separation efficiency was determined by photoluminescence (PL) spectroscopy. The intensity of the steady‐state PL signal gradually decreases from Cage‐PorCOF to Cage‐PorCOF(Ni) and further to Cage‐PorCOF(Co) (Figure 6c), which suggests that the incorporation of atomic Co or Ni into the COF skeletons can improve the charge transfer, resulting in more efficient charge separation.^[^ ^72^ ^]^ The transient photocurrent density of Cage‐PorCOF(Co) is higher than that of Cage‐PorCOF(Ni) and Cage‐PorCOF (Figure 6d), further illustrating more effective charge separation in Cage‐PorCOF(Co).^[^ ^34^ ^]^ Time‐resolved PL (TRPL) spectrum of Cage‐PorCOF(Co) shows a shorter carrier lifetime (1.40 ns) compared with those of Cage‐PorCOF (1.63 ns) and Cage‐PorCOF(Ni) (1.43 ns) (Figure 6e), which indicates that photoexcited electrons are quickly captured by CO_2_ on the Co active centers of Cage‐PorCOF(Co) to boost the photocatalytic CO_2_ reduction, with effectively accelerated charge separation and transfer.^[^ ^73^ ^]^ Compared with Cage‐PorCOF and Cage‐PorCOF(Ni), Cage‐PorCOF(Co) exhibits the smallest semicircle in the Nyquist plot (Figure 6f), which indicates that Cage‐PorCOF(Co) has the lowest charge transfer resistance and the best charge transport and separation efficiency.^[^ ^9^ ^]^ Femtosecond transient absorption spectroscopy (Fs‐TAS) was utilized to investigate the charge transfer dynamics in both Cage‐PorCOF(Co) and Cage‐PorCOF(Ni) (Figures 6g–i and S32). The spectra exhibited broad negative bleaching signals spanning from 410 to 540 nm, which were attributed to ground‐state bleaching (GSB). Subsequently, as the delay time increased, the negative bleaching signals gradually transformed into excited‐state absorption signals (ESA), indicating the occurrence of charge transfer (Figure 6g,h). Notably, the signal changes in Cage‐PorCOF(Co) were more pronounced than those in Cage‐PorCOF(Ni). As depicted in Figure 6i, the decay kinetic curves of the two metalated COFs were analyzed at a wavelength of 520 nm. The average lifetime of Cage‐PorCOF(Co) was calculated to be 511.48 ps, which was substantially longer than that (222.06 ps) of Cage‐PorCOF(Ni). This result suggests that the charge carrier photogenerated in Cage‐PorCOF(Co) has a much longer lifetime and a more stable separated state compared to Cage‐PorCOF(Ni). Based on all of these characterizations, high catalytic activity of Cage‐PorCOF(Co) should be attributed to the synergistic effects of the atomic dispersion of metallic catalytic sites, CO_2_‐affinity of the frameworks, and incorporated metal atoms, as well as the light absorption and charge separation ability of metalloporphyrin groups.

**Figure 7:**
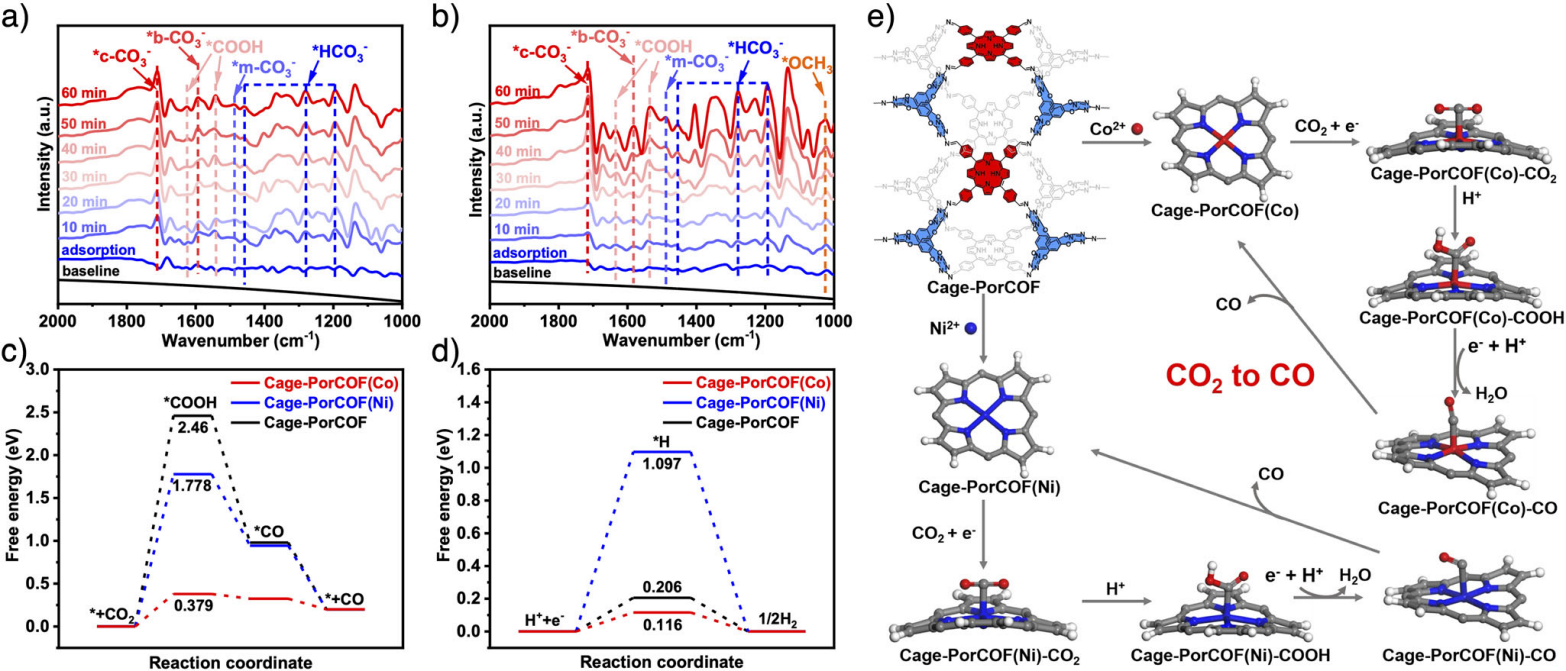
In situ DRIFT measurements for CO_2_ photoreduction over a) Cage‐PorCOF(Co) and b) Cage‐PorCOF(Ni) collected at different time intervals upon light illumination. DFT‐calculated Gibbs free energy (Δ*G*, eV) profiles for c) photoreduction of CO_2_ to CO and d) hydrogen evolution reaction on Cage‐Por‐COF, Cage‐PorCOF(Co), and Cage‐PorCOF(Ni). e) Proposed mechanism regarding the photocatalytic CO_2_ reduction over Cage‐PorCOF(Co) and Cage‐PorCOF(Ni).

Subsequently, the mechanism of the CO_2_ photoreduction reaction was investigated by spectral measurements combined with density functional theory (DFT) calculations. The in situ diffuse reflectance infrared Fourier transform spectroscopy (DRIFTS) measurements were carried out with Cage‐PorCOF(Co) and Cage‐PorCOF(Ni) as photocatalysts to detect the reaction intermediates in the process of CO_2_ photoreduction. The characteristic peaks at 2339 and 2369 cm^−1^ for Cage‐PorCOF(Co) and 2341 and 2371 cm^−1^ for Cage‐PorCOF(Ni) were clearly observed after the introduction of CO_2_ (Figures S33 and S34), which should be attributed to the stretching vibration of the CO_2_ molecule adsorbed on the surface of the photocatalysts.^[^ ^74^, ^75^ ^]^ As shown in Figure 7a, several carboxylate complexes were detected from the in situ DRIFTS of Cage‐PorCOF(Co). According to literature reports,^[^ ^76^ ^]^ the peaks at 1487 and 1593 cm^−1^ correspond to the monodentate carbonate species (*m*‐CO_3_^2−^) and the bidentate carbonate species (*b*‐CO_3_^2−^) respectively, whereas the peaks at 1711 cm^−1^ is ascribed to the chelating bridged carbonate species (*c*‐CO_3_^2−^). The peaks at 1194, 1279, and 1458 cm^−1^ belong to *CO_2_‐intermediates, symmetric stretching of *HCO_3_^−^, and asymmetric stretching of *HCO_3_^−^, respectively.^[^ ^9^, ^76^ ^]^ All the detected carboxylate complex species are reaction intermediates for CO_2_‐to‐CO production. In addition, the characteristic peaks at 1542 and 1624 cm^−1^ can be assigned to *COOH, which is an important reaction intermediate of CO production for revealing the key transformation process of CO_2_ in the photocatalytic reaction.^[^ ^76^ ^]^ From the in situ DRIFTS of Cage‐PorCOF(Ni) (Figure 7b), besides the above characteristic peaks, the peak at 1025 cm^−1^ being attributed to *OCH_3_ was also observed, which is consistent well with the result of trace CH_4_ generation by Cage‐PorCOF(Ni).^[^ ^27^ ^]^ The absorption peaks of all the detected intermediates become stronger gradually upon prolonging the irradiation time, implying the progress of the photocatalytic reaction.

To elucidate the underlying mechanisms behind the enhanced catalytic performance of Cage‐PorCOF(Co) and to gain deeper insight into the nature of the active catalytic sites, DFT calculations were also conducted. As depicted in Figure 7c, the CO_2_ reduction pathways and intermediate species of Cage‐PorCOF, Cage‐PorCOF(Co), and Cage‐PorCOF(Ni) follow identical processes, involving *+CO_2_, *COOH, *CO, and *+CO intermediates. Among these steps, the formation of *COOH serves as the rate‐determining step in the photoreduction of CO_2_ to CO as it possesses the highest energy barrier.^[^ ^71^ ^]^ The Gibbs free energy (Δ*G*) value for *COOH formation in Cage‐PorCOF(Co) is 0.379 eV, lower than that of both Cage‐PorCOF (Δ*G* = 2.46 eV) and Cage‐PorCOF(Ni) (Δ*G* = 1.778 eV). This observation suggests that the incorporation of Co effectively optimizes the rate‐determining step, thereby accelerating the reaction kinetics of CO_2_ reduction. Subsequently, the transition from *COOH to *CO in both Cage‐based COFs is thermodynamically favorable owing to its downhill energy barriers.^[^ ^72^ ^]^ More importantly, Cage‐PorCOF(Co) presents a lower energy barrier for the transition from *CO to *+CO compared to Cage‐PorCOF and Cage‐PorCOF(Ni). Consequently, CO is more readily produced on the active sites of Cage‐PorCOF(Co).^[^ ^71^ ^]^ The Gibbs free energy profiles for the H_2_ evolution reaction (HER) on Cage‐PorCOF, Cage‐PorCOF(Co), and Cage‐PorCOF(Ni) were also calculated and illustrated in Figure 7d. The absorption of protons to generate *H is identified as the rate‐determining step in the successive HER pathway, with energy barriers of 0.206, 0.116, and 1.097 eV for Cage‐PorCOF, Cage‐PorCOF(Co), and Cage‐PorCOF(Ni), respectively. Notably, Cage‐PorCOF(Ni) displays much higher barriers for *H formation compared to Cage‐PorCOF and Cage‐PorCOF(Co), leading to a remarkable selectivity for CO_2_ reduction to CO and the inhibition of H_2_ evolution from water splitting.^[^ ^17^ ^]^

Based on the experimental results and theoretical findings, a plausible mechanism for the photoreduction of CO_2_ to CO catalyzed by Cage‐PorCOF(Co) and Cage‐PorCOF(Ni) was proposed in Figure 7e. Taking Cage‐PorCOF(Co) as an example, under visible light irradiation, the photosensitizer [Ru(bpy)_3_]Cl_2_ is excited, facilitating the transfer of photogenerated electrons to the Co sites in the Cage‐PorCOF(Co) catalyst. Meanwhile, the resulting holes are scavenged by the electron donor TEOA, which is oxidized to TEOA^·+^, thus completing the catalytic cycle. The incorporation of Co ions in Cage‐PorCOF(Co) enhances electron‐hole separation, promoting the photocatalytic reduction of adsorbed CO_2_ to CO.

## Conclusion

In summary, a highly crystalline and porous 3D COF with pto topology has been rationally designed and successfully constructed based on the Schiff‐base condensation of the triple‐symmetrical nitrogen‐rich supramolecular cage and planar quadruple‐symmetrical photoactive porphyrin‐based reactants. Subsequently, two metalated COFs (Cage‐PorCOF(M), M = Co or Ni) have been achieved by the simple postmetalation processes. By featuring highly porous 3D structure, the incorporation of the CO_2_‐affinity nitrogen‐rich cages and the light absorbing porphyrin moieties, as well as the fully exposed single‐atomic metal sites, Cage‐PorCOF(M) has been employed for the CO_2_ photoreduction. Cage‐PorCOF(Co), as a promising photocatalyst, shows a high CO production rate of up to 48 748 µmol g^−1^ h^−1^ and the selectivity as high as 97.9% within the first initiating hour, a long‐time continuous CO generation capability, and a preeminent cycling durability, which is superior to the most reported COF‐based photocatalysts for CO_2_ photoreduction. Cage‐PorCOF(Ni) also exhibits a high CO generation rate of up to 28 446 µmol g^−1^ h^−1^ and nearly 100% selectivity within the first initiating hour, and a long‐time continuous and recyclable ability. Comprehensive experimental characterizations and theoretic calculations indicate the important roles of nitrogen‐rich cage and porphyrin moieties, the single atomic metal sites as well as the highly porous frameworks in the photocatalytic CO_2_ reduction, where the nitrogen‐rich cage enhances the adsorption of CO_2_, the porphyrin moieties broaden the light absorption range, and fully exposed atomic Co active sites in the 3D COF activate the adsorbed CO_2_ molecule, promoting the charge transfer kinetics and finally enhancing the catalytic activity. By the integration of multiple functionalities into porous frameworks, this work provides a reliable way to synthesize effective photocatalysts for CO_2_ conversion.

## Conflict of Interests

The authors declare no conflict of interest.

## Supporting information



Supporting Information

## Data Availability

The data that support the findings of this study are available from the corresponding author upon reasonable request.
