# Supplementary material for: Integrating Multifunctionalities into a 3D Covalent Organic Framework for Efficient CO2 Photoreduction
Source: Angew Chem Int Ed Engl. 2025 May 2;64(26):e202504772. doi: 10.1002/anie.202504772 (PMC12184286; doi:10.1002/anie.202504772)
Supplement: Supplementary file 1 — Supporting Information [file ANIE-64-e202504772-s001.docx]

**Supporting Information**

**1. Supporting experimental procedures**

**General information.** All starting materials are commercially available and were used as received unless specifically mentioned: acetic acid (AR, Acmec), acetone (AR, Kangde), acetonitrile (AR, sinopharm), ammonium hydroxide (AR, Macklin), aniline (AR, Aladdin), cobalt (II) acetate tetrahydrate (99%, bidepharm), cyanuric chloride (≥99%, Acmec), N,N-diisopropylethylamine (≥99%, Aladdin), 1,4-dioxane (AR, Acmec), mesitylene (AR, Macklin), nickel (II) acetate tetrahydrate (99%, bidepharm), phloroglucinol (≥98%, Acmec), tetrahydrofuran (AR, Macklin), 5,10,15,20-tetrakis(4-benzaldehyde)porphyrin (≥95%, Alfa), tris(2,2'-bipyridyl)dichlororuthenium(II) hexahydrate (98%, Macklin), and triethanolamine (AR, DAMAO).

Powder X-ray diffraction (PXRD) data were collected at 40 kV, 200 mA on a SmartLab 9 kW X-Ray diffractometer using Cu-K*α* radiation (*λ* = 1.5418 Å) over 2θ range of 2.0° - 30.0° at room temperature. Thermogravimetric analyses (TGA) were carried out on a Netzsch TG 209 F3 Tarsus with a heating rate of 10 °C min^-1^ under N_2_ flow (60 mL/min) from room temperature to 800 °C. Surface area and pore size distribution were analyzed by N_2_ adsorption and desorption using a Kubo-X1000 at 77 K. ^1^H NMR spectra were measured on a Bruker AVANCE-400 spectrometer (400 MHz ^1^H) at ambient temperature. All chemical shifts (δ) are quoted in ppm. Solid-state ^13^C (150.03 MHz) CP-MAS NMR spectra were performed on a Bruker WB 600 spectrometer at 300 K with a 3.2 mm double channel probehead. Samples were carefully packed into a 3.2 mm cylindrical zirconia rotor with Kel-F caps. ^13^C NMR spectra were originally calibrated with adamantane as the standard sample. Scanning electron microscopy (SEM) images were obtained on a Zeiss scanning electron microscope. Transmission electron microscopy (TEM) images were recorded using a FEI talos F200s. Fourier Transform infrared spectroscopy (FT-IR) was recorded on a Bruker Tensor II. X-ray photoelectron spectroscopy (XPS) was recorded on PHI 5000 Versaprobe III. Steady-state fluorescence spectra and time-resolved photoluminescence spectra were carried out using an Edinburgh FLS1000 photoluminescence spectrometer. UV-Vis diffuse reflection absorption spectra of samples were detected by Hitachi UH4150 equipped with an integration sphere within the wavelength range of 200-800 nm. In situ DRIFTS measurements were conducted using the Bruke Tensor II FTIR NEXUS spectrometer equipped with an in situ diffuse reflectance cell. A 300 W Xe lamp (CEL-PF300-T6, Beijing China Education Au-light Technology Co., Ltd) was adopted as the light source. The production rates of CO, CH_4_ and H_2_ were analyzed by gas chromatography (GC-7920).

**1,3,5-Tris((4,6-dichloro-1,3,5-triazin-2-yl)oxy)benzene (3)** was prepared according to the published procedure.^[S1]^ A mixture of **1** (2 g, 15.9 mmol) and DIPEA (7.82 g, 60.5 mmol) in THF (60 mL) was added dropwise over a period of 30 min to an ice-cooled and well-stirred solution of **2** (13.16 g, 71.4 mmol) in THF (100 mL). The reaction was kept stirring at 0 °C for 4 h. After that, the reaction mixture was extracted with CH_2_Cl_2_ (50 mL × 2). The combined organic layer was washed with water (200 mL × 2), dried over anhydrous Na_2_SO_4_ and then evaporated under reduced pressure. The crude product was further purified using a column chromatograph (CH_2_Cl_2_/n-Hexane, 100/20) to give compound **3** as a white solid (6.5 g, 11.4 mmol, yield: 72%). ^1^H NMR (400 MHz, Chloroform-*d*) δ 7.16 (s, 3H).

**Scheme S1.** Schematic synthesis of **Cage 4**.

**Cage 4** was prepared according to the published procedure.^[S1]^ Solutions of **1** (221 mg, 1.8 mmol) in acetone (75 mL) and **3** (1 g, 1.8 mmol) in acetone (75 mL) were added dropwise at the same rate to a solution of DIPEA (0.821 g, 3.6 mmol) in acetone (150 mL) at room temperature over a period of 4 h. After addition, the resulting mixture was kept stirring at room temperature for another 3 days. The reaction mixture was extracted with CH_2_Cl_2_ (50 mL × 2). The combined organic layer was washed with water (200 mL × 2), dried over anhydrous Na_2_SO_4_ and then evaporated under reduced pressure. The crude product was further purified using a column chromatograph (CH_2_Cl_2_/CH_3_COOC_2_H_5_, 100/2) to give **cage 4** as a white solid (0.37 g, 0.63 mmol, yield: 35%). ^1^H NMR (400 MHz, Chloroform-*d*) δ 6.69 (s, 6H).


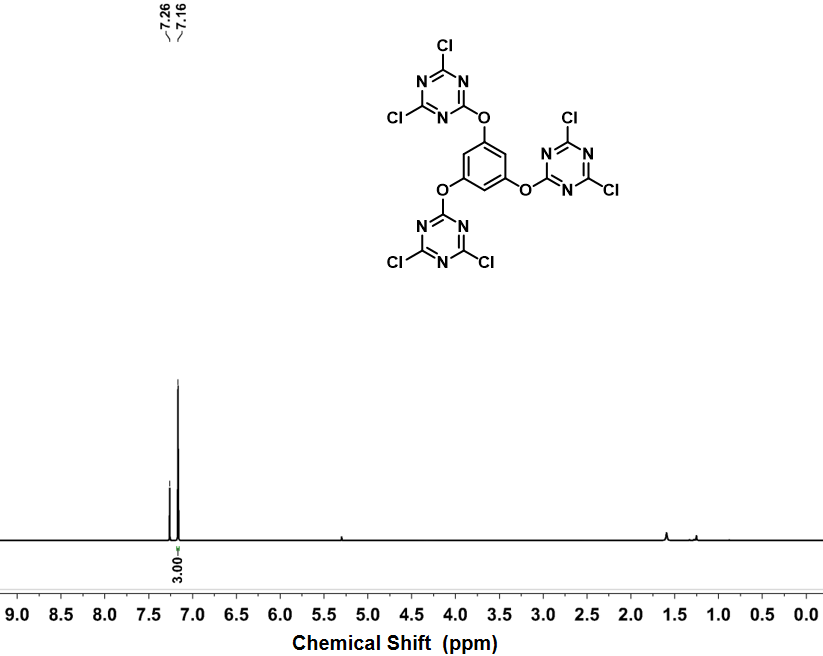


^1^H NMR (400 MHz, CDCl_3_) spectrum of compound **3**.


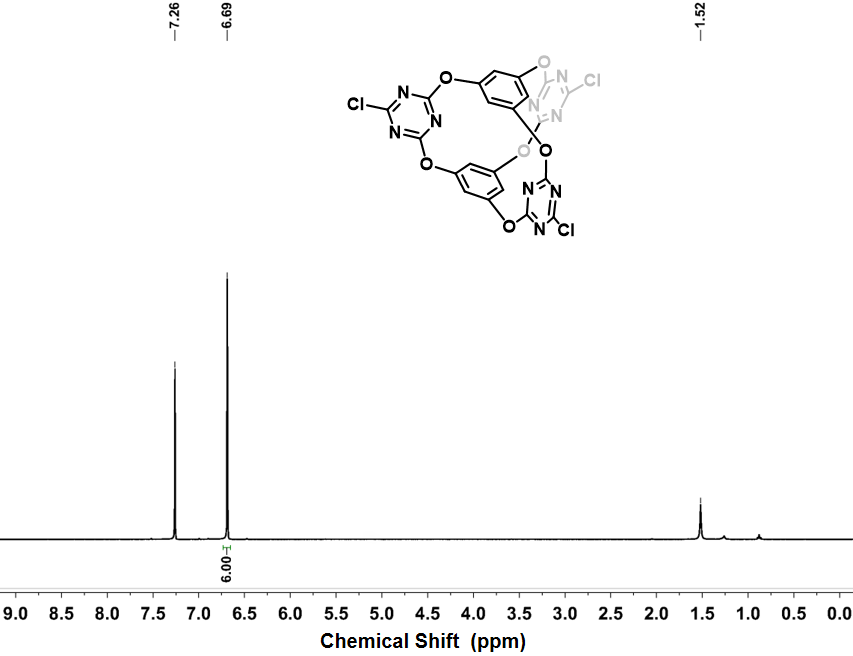


^1^H NMR (400 MHz, CDCl_3_) spectrum of **cage 4**.

**Cage-3NH_2_** was prepared according to the published procedure.^[S2,S3]^ To a solution of **4** (585 mg, 1.00 mmol) in acetone (30 mL), ammonium hydroxide (400 μL, 28%) was added. The resulting mixture was stirred at room temperature for 24 h. The white solid was obtained by filtration and washing with acetone (20 mL × 3) and water (20 mL × 3). The product **Cage-3NH_2_** was dried under vacuum at 60 °C and obtained with the yield of 80 % (410 mg). ^1^H NMR (DMSO-*d*_6_, 400 MHz): *δ* (ppm) 7.77 (s, 6H), 6.71 (s, 6H).

**Scheme S2.** Schematic synthesis of **Cage-3NH_2_**.


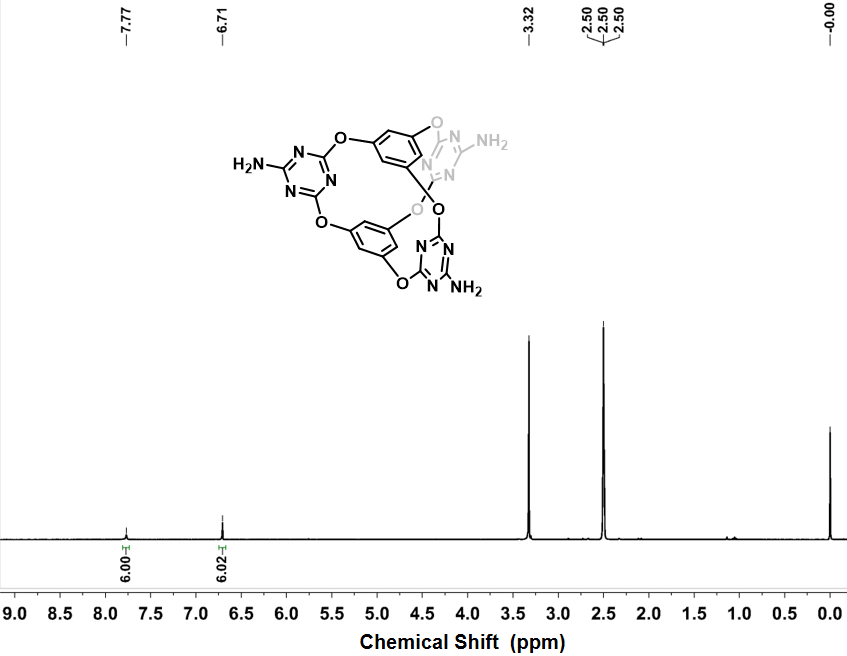


^1^H NMR (400 MHz, DMSO-*d*_6_) spectrum of **Cage-3NH_2_**.

**Synthesis of Cage-PorCOF.** A 10 mL Schlenk tube was charged with Cage-3NH_2_ (15.9 mg, 0.03 mmol), 5,10,15,20-tetrakis(4-benzaldehyde) porphyrin (*p*-Por-CHO, 16.4 mg, 0.023 mmol), 1,4-dioxane (0.2 mL), mesitylene (0.8 mL) and aniline (12.3 μL, 0.135 mmol). The resulting mixture was sonicated for 15 min. Acetic acid (6 M, 0.3 mL) was then added to the solution, and the Schlenk tube was flash frozen at 77 K using the liquid nitrogen bath, evacuated and sealed by Teflon valve. Upon warming to room temperature, the Schlenk tube was heated at 120 °C for 5 days. Purple solid at the bottom of the tube was isolated by hot filtration and washed with DMF (10 mL) and THF (10 mL). The obtained powder was washed by Soxhlet extractions with THF for 48 h. The powder was dried at 60 ºC under vacuum overnight to afford **Cage-PorCOF** as purple powder in 70% yield.


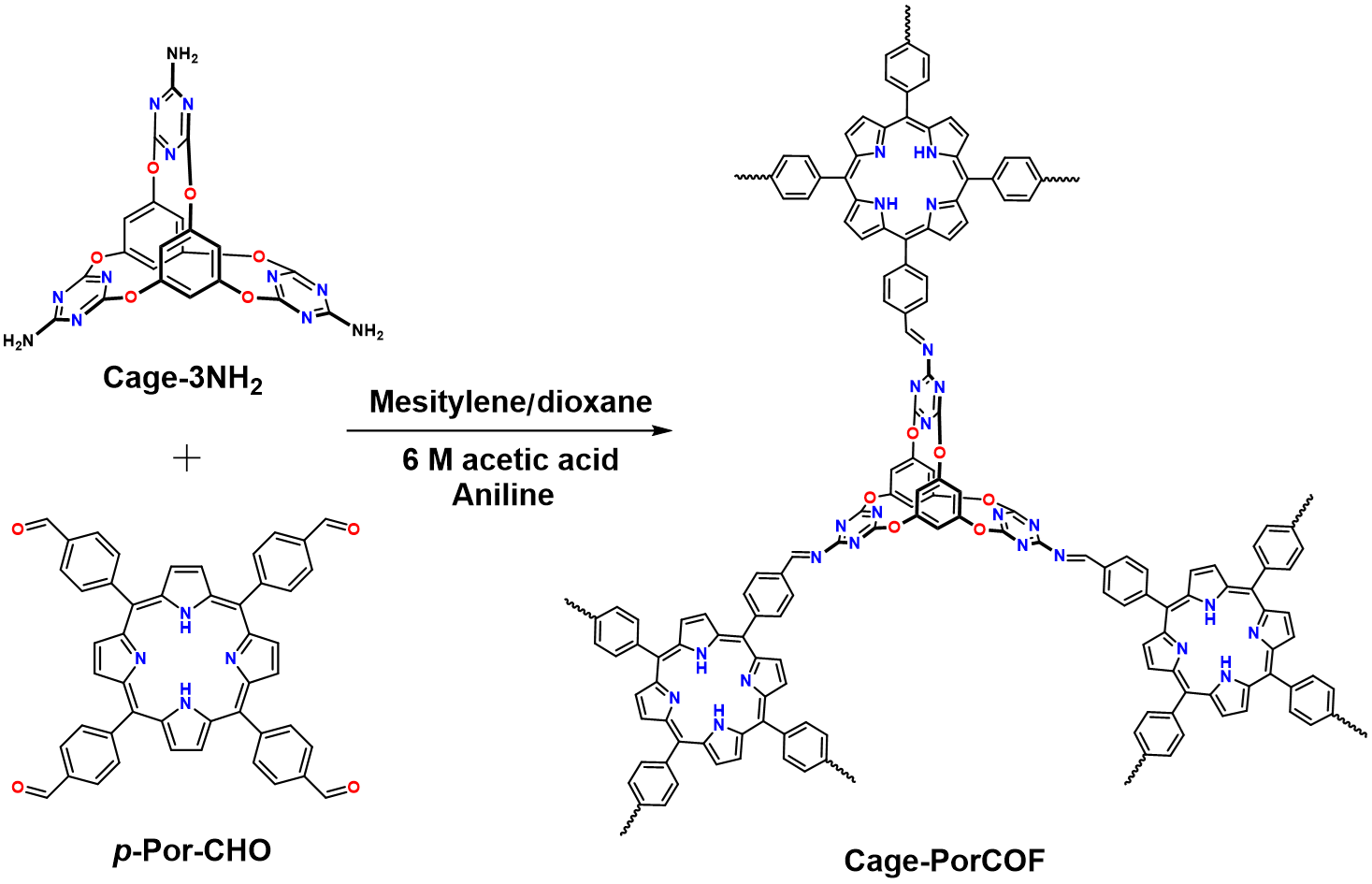


**Scheme S3.** Schematic synthesis of **Cage-PorCOF**.

**Synthesis of Cage-PorCOF(Co) and Cage-PorCOF(Ni).** **Cage-PorCOF(Co)** and **Cage-PorCOF(Ni)** were synthesized by a post synthesis method.^[S4]^ In detail, **Cage-PorCOF** (15 mg) and Co(OAc)_2_·4H_2_O (50 mg) or Ni(OAc)_2_·4H_2_O (50 mg) were added to the ethanol (20 mL). After being purified by N_2_, the mixture was heated and refluxed at N_2_ atmosphere for 12 h. Following that, the solutions were cooled down to room temperature and filtered. The filter cake was washed thoroughly with water and ethanol to remove free metal ions. The final filter cake was dried at 120 °C under dynamic vacuum overnight to get **Cage-PorCOF(Co)** or **Cage-PorCOF(Ni)**.

**Structural modeling and PXRD Analysis.** Molecular modeling of COFs was generated with the Materials Studio (ver. 2017) suite of programs.^[S5]^ Pawley refinement was carried out using Reflex, a software package for crystal determination from PXRD pattern. Unit cell dimension was set to the theoretical parameters. The Pawley refinement was performed to optimize the lattice parameters iteratively until the R*wp* value converges and the overlay of the observed with refined profiles shows good agreement. The lattice models (e.g., cell parameters, atomic positions, and total energies) were then fully optimized using MS Forcite molecular dynamics module (universal force fields, Ewald summations) method.

**CO_2_ photoreduction measurement.** The custom-made reactor for the photocatalytic CO_2_ reduction was established in Pyrex glass. Typically, COFs (10 mg) and photosensitizer [Ru(bpy)_3_]Cl_2_·6H_2_O (10 mg) were dispersed into a solution of acetonitrile/H_2_O/triethanolamine (12 mL, *v*:*v:v* = 4:1:1) in the reactor. After the air was completely emptied, CO_2_ gas was infused into the quartz reactor. This process was repeated for three times. After the last cycle, the flask was backfilled with CO_2_ (1.0 atm). The temperature of the reaction solution was maintained at 25 °C and the reaction mixture was irradiated under Xe lamp with full-wave band light. After reaction, the gaseous products were analyzed by gas chromatography (GC-7920). H_2_ was analyzed by a thermal conductivity detector (TCD). CO and CH_4_ were detected by a flame ionization detector (FID). The cyclic tests were conducted by directly recovering the used catalysts and then re-dispersed them into a fresh solution for cycling tests. The isotope labeling experiments were performed using ^13^CO_2_ feeding gas instead of ^12^CO_2_, and the products were analyzed by gas chromatography-mass spectrometers (Agilent, 7890A and 5975C).

**Photoelectrochemical measurement.** Photoelectrochemical and electrochemical measurements including photocurrent, electrochemical impedance spectra (EIS), and Mott-Schottky plot were performed with an electrochemical working station (CHI 760E) via a three-electrode cell. The catalyst sample was electroplated on the ITO glass to prepare the working electrode, while Ag/AgCl electrode and Pt plate electrode were immersed in sodium sulfate aqueous solution (0.5 M) to serve as the reference electrode and the counter electrode, respectively. Transient photocurrent response with an interval of 20 s on/off switching was measured under visible light. EIS was carried out in the frequency range of 0.1 Hz - 100 KHz with an AC amplitude of 5 mV under visible light. For Mott-Schottky plot measurements, the samples were tested with different frequencies at 1000, 1500, and 2000 Hz.

**Apparent quantum efficiency.** The apparent quantum efficiency (AQE) of the catalysts was measured by different bandpass filters (including 380 nm, 420 nm, 450 nm, 475 nm, 550 nm, 550 nm and 600 nm) under the same photocatalytic reaction conditions, and the light intensity was detected by a CEL-NP2000 optical power meter (Beijing China Education Au-light Technology Co., Ltd). The AQE values were calculated by the equation:^[S6]^ AQE (%) = (Total consumed electron number/number of incidented photon number) ×100% = (2×*n*_co_×N_A_×h×c)/(S×*P*×t×λ) ×100%, where *n*_co_ is the molar number of the CO, N_A_ is Avogadro’s constant (6.022×10^23^ mol^-1^), h is the Planck’s constant (6.626×10^-34^ J·s^-1^), c is the speed of light (2.998×10^8^ m·s^-1^), S is the irradiation area (cm^2^), *P* is irradiation intensity (W cm^-2^), t is irradiation time (s), and λ is the wavelength of the monochromatic light, respectively.

**Computational details.** We employed the Vienna Ab Initio Package (VASP)^[S7,S8]^ to perform all spin-polarized density functional theory (DFT) calculations within the generalized gradient approximation (GGA) using the Perdew-Burke-Ernzerhof (PBE)^[S9]^ formulation. We chose the projected augmented wave (PAW) potentials^[S10,S11]^ to describe the ionic cores and take valence electrons into account using a plane wave basis set with a kinetic energy cutoff of 450 eV. Partial occupancies of the Kohn-Sham orbitals were allowed using the Gaussian smearing method and a width of 0.05 eV. The electronic energy was considered self-consistent when the energy change was smaller than 10^-5^ eV. A geometry optimization was considered convergent when the energy change was smaller than 0.02 eV Å^-1^. The vacuum spacing in a direction perpendicular to the plane of the structure is 18 Å. The weak interaction was described by DFT+D3 method using empirical correction in Grimme’s scheme.^[S12,S13]^ The Gibbs free energy change (ΔG) of each chemical reaction was calculated by equation ΔG = ΔE + ΔZPE – TΔS, where E is the calculated total energy, ZPE is the zero-point energy, T is the temperature, and S is the entropy.

**2. Supporting Tables**

**Table S1.** Fractional atomic coordinates for **Cage-PorCOF**.

| Space group: *P-42c*  *a* = *b* = 44.6112 Å, *c* = 45.6209 Å; *α* = *β* = *γ* = 90º | | | |
| --- | --- | --- | --- |
| C1 | -0.24772 | 0.5634 | 0.26489 |
| N2 | -0.25059 | 0.5449 | 0.24349 |
| C3 | -0.24613 | 0.56019 | 0.21995 |
| C4 | -0.23993 | 0.58801 | 0.22653 |
| C5 | -0.24107 | 0.59007 | 0.25499 |
| C6 | -0.24912 | 0.54992 | 0.19244 |
| C7 | -0.24768 | 0.57039 | 0.16891 |
| C8 | -0.22516 | 0.56933 | 0.14986 |
| C9 | -0.22354 | 0.58882 | 0.12797 |
| C10 | -0.24422 | 0.60976 | 0.12499 |
| C11 | -0.26688 | 0.61074 | 0.14396 |
| C12 | -0.26865 | 0.59108 | 0.16568 |
| C13 | -0.24147 | 0.63041 | 0.10215 |
| N14 | -0.25853 | 0.65166 | 0.09983 |
| H15 | -0.23499 | 0.60455 | 0.20861 |
| H16 | -0.23762 | 0.60909 | 0.27074 |
| H17 | -0.2073 | 0.55181 | 0.15222 |
| H18 | -0.20454 | 0.58758 | 0.11187 |
| H19 | -0.28468 | 0.62832 | 0.14163 |
| H20 | -0.28801 | 0.592 | 0.18138 |
| C21 | 0.06072 | -0.01907 | -0.00417 |
| N22 | 0.04241 | 0.00253 | -0.00011 |
| C23 | 0.05801 | 0.02636 | -0.0027 |
| C24 | 0.08588 | 0.01985 | -0.00875 |
| C25 | 0.08758 | -0.00892 | -0.00977 |
| C26 | 0.04795 | 0.05405 | 0.00153 |
| C27 | 0.06897 | 0.07727 | 0.00352 |
| C28 | 0.07128 | 0.0975 | -0.01781 |
| C29 | 0.09168 | 0.11881 | -0.01611 |
| C30 | 0.11006 | 0.12002 | 0.00694 |
| C31 | 0.10755 | 0.09993 | 0.02829 |

**Table S2.** Comparison of CO_2_ photoreduction performance catalyzed by various metal-supported porous organic materials.

| Catalyst | Light Condition | CO  (*μ*mol g^-1^ h^-1^) | Sel. (%) | Ref. |
| --- | --- | --- | --- | --- |
| **Cage-PorCOF(Co)** | 300 W Xe lamp  (full-wave band) | 48748 (1 h) | 97.9 | This work |
|  |  | 23295 (4 h) | 98.3 |  |
| **Cage-PorCOF(Ni)** |  | 28446 (1 h) | 99.95 |  |
|  |  | 11081 (4 h) | 99.97 |  |
| Co-Btt-Bpy COF | 10 W LED lamp  (λ = 420 nm) | 9800 | 78.7 | [S14] |
| MCOF-Co-315 | 300 W Xe lamp  (λ > 370 nm) | 1616 | - | [S15] |
| TOT-TAPP | 300 W Xe lamp  (800 > λ > 420 nm) | 34.8 | - | [S16] |
| USTB-11(Cu,Ni) | 300 W Xe lamp  (λ > 420 nm) | 22130 | 98 | [S17] |
| Co-2,3-DHTA-COF | 300 W Xe lamp  (λ > 420 nm) | 18000±700 | 95.7 | [S18] |
| JUC-640-Co | 300 W Xe lamp  (λ > 380 nm) | 15139 | 94.4 | [S19] |
| Re-COF | 225 W Xe lamp  (λ > 420 nm) | 15000 | 98 | [S20] |
| COF-367-Co NSs | 300 W Xe lamp  (λ > 420 nm) | 10162 | 78 | [S21] |
| CdS/TpBpy-20% | Simulated solar light (AM 1.5G) | 8800 | 85 | [S22] |
| H-COF-Ni | 300 W Xe lamp  (λ > 420 nm) | 5694 | 96 | [S23] |
| NiPc-CoPOP | White LED  (400 nm-800 nm) | 4270 | 54 | [S24] |
| Ni-TpBpy | 300 W Xe lamp  (λ > 420 nm) | 4057 | 96 | [S25] |
| NiTCPE-*pstp* | 300 W Xe lamp  (λ > 420 nm) | 3353.6 | 99.95 | [S26] |
| HOF-25-Re | 300 W Xe lamp  (λ > 420 nm) | 3030 | 92 | [S27] |
| Co-OAc | 300 W Xe lamp  (λ > 380 nm) | 2325.7 | 99.1 | [S28] |
| pNJU-319Fe | 300 W Xe lamp  (λ > 420 nm) | 688 | 90 | [S29] |

**Table S3.** EXAFS fitting parameters at the Co K-edge for various samples.

| Sample | Shell | *CN^a^* | *R*(Å)*^b^* | *σ*^2^(Å^2^)*^c^* | Δ*E*_0_(eV)*^d^* | *R* factor |
| --- | --- | --- | --- | --- | --- | --- |
| Co foil | Co-Co | 12* | 2.49±0.01 | 0.0071 | 7.7 | 0.0055 |
| CoO | Co-O | 6* | 2.13±0.01 | 0.0098 | 0.9 | 0.0106 |
|  | Co-Co | 12* | 3.01±0.01 | 0.0080 | -3.0 |  |
| CoPc | Co-N | 4* | 1.89±0.01 | 0.0025 | 3.0 | 0.0129 |
| **Cage-PorCOF(Co)** | **Co**-**N** | **4.0±0.6** | **1.95±0.01** | **0.0023** | **0.1** | **0.0157** |

*^a^CN*, coordination number; *^b^R*, distance between absorber and backscatter atoms; *^c^σ*^2^, Debye-Waller factor to account for both thermal and structural disorders; *^d^ΔE*_0_, inner potential correction; *R* factor indicates the goodness of the fit. S_0_^2^ was fixed to 0.798, according to the experimental EXAFS fit of Co foil by fixing CN as the known crystallographic value. Fitting range: 2.0 ≤ *k* (/Å) ≤ 12.0 and 1.4 ≤ *R* (Å) ≤ 2.8 (Co foil); 2.0 ≤ *k* (/Å) ≤ 12.0 and 1 ≤ *R* (Å) ≤ 3.0 (CoO); 2.0 ≤ *k* (/Å) ≤ 12.0 and 1.0 ≤ *R* (Å) ≤ 1.9 (CoPc); 2.0 ≤ *k* (/Å) ≤ 12.0 and 1.0 ≤ *R* (Å) ≤ 2.0 (Cage-PorCOF(Co)). A reasonable range of EXAFS fitting parameters: 0.600 < *Ѕ*_0_^2^ < 1.000; *CN >* 0; *σ*^2^ > 0 Å^2^; |Δ*E*_0_| < 15 eV; *R* factor < 0.02.

**3. Supporting Figures**


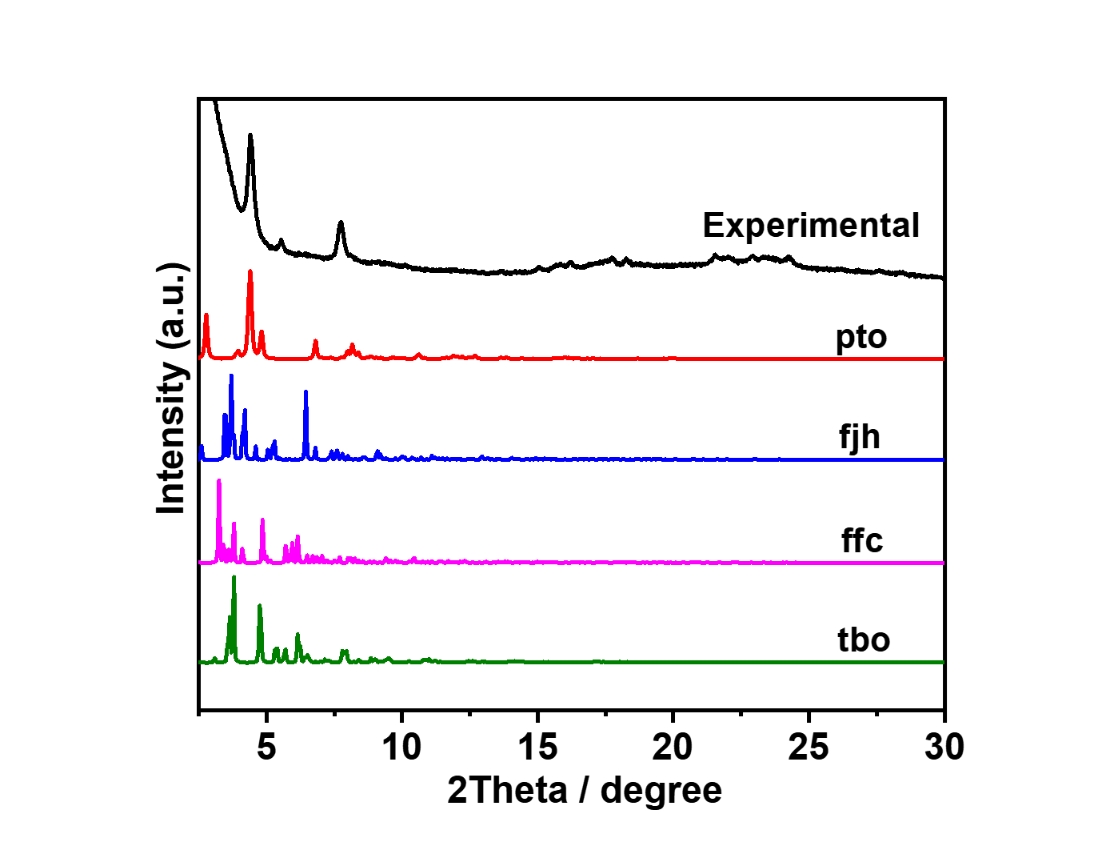


**Figure S1**. Experimental and simulated PXRD patterns of **Cage-PorCOF**.


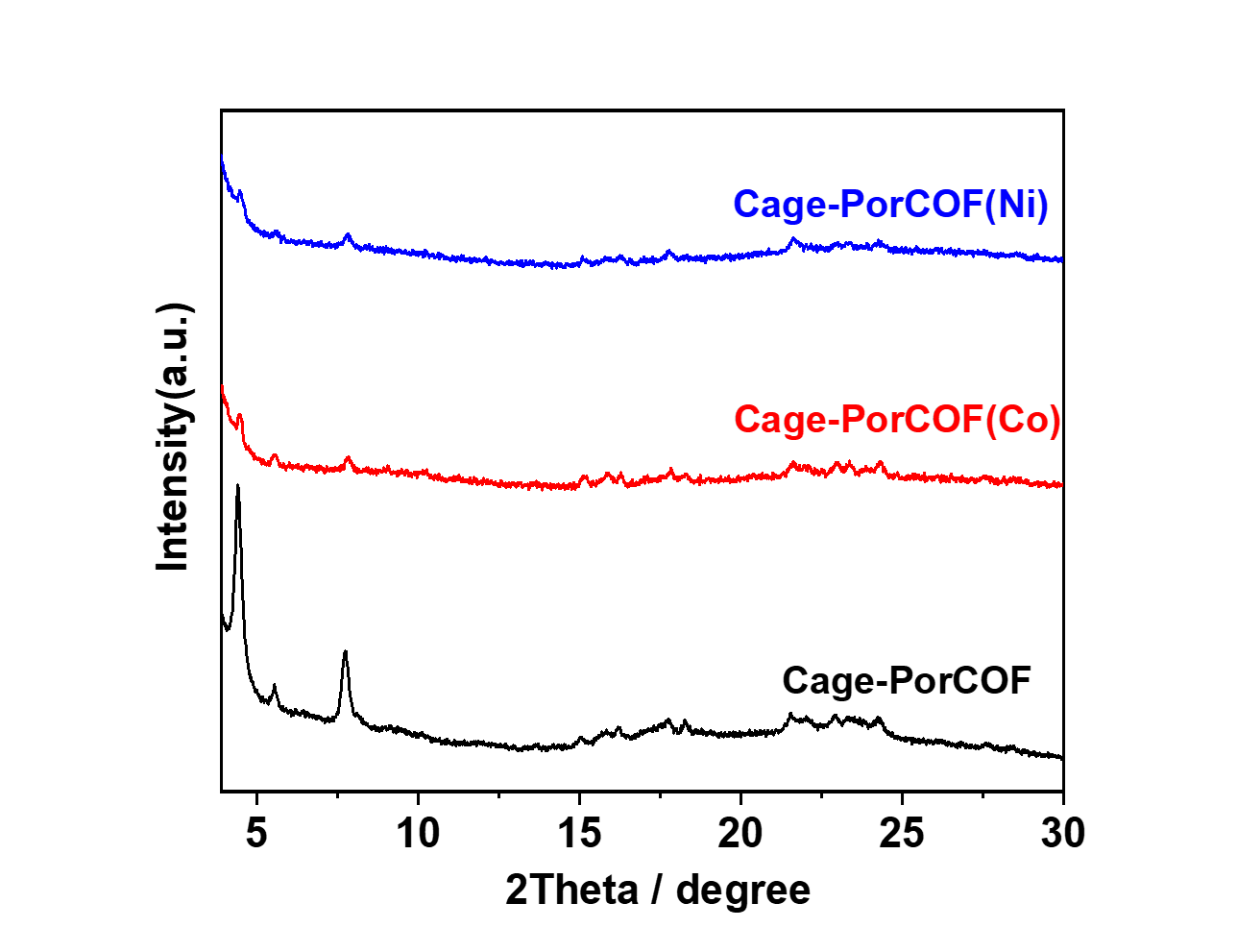


**Figure S2**. PXRD patterns of **Cage-PorCOF**, **Cage-PorCOF(Co)** and **Cage-PorCOF(Ni)**.


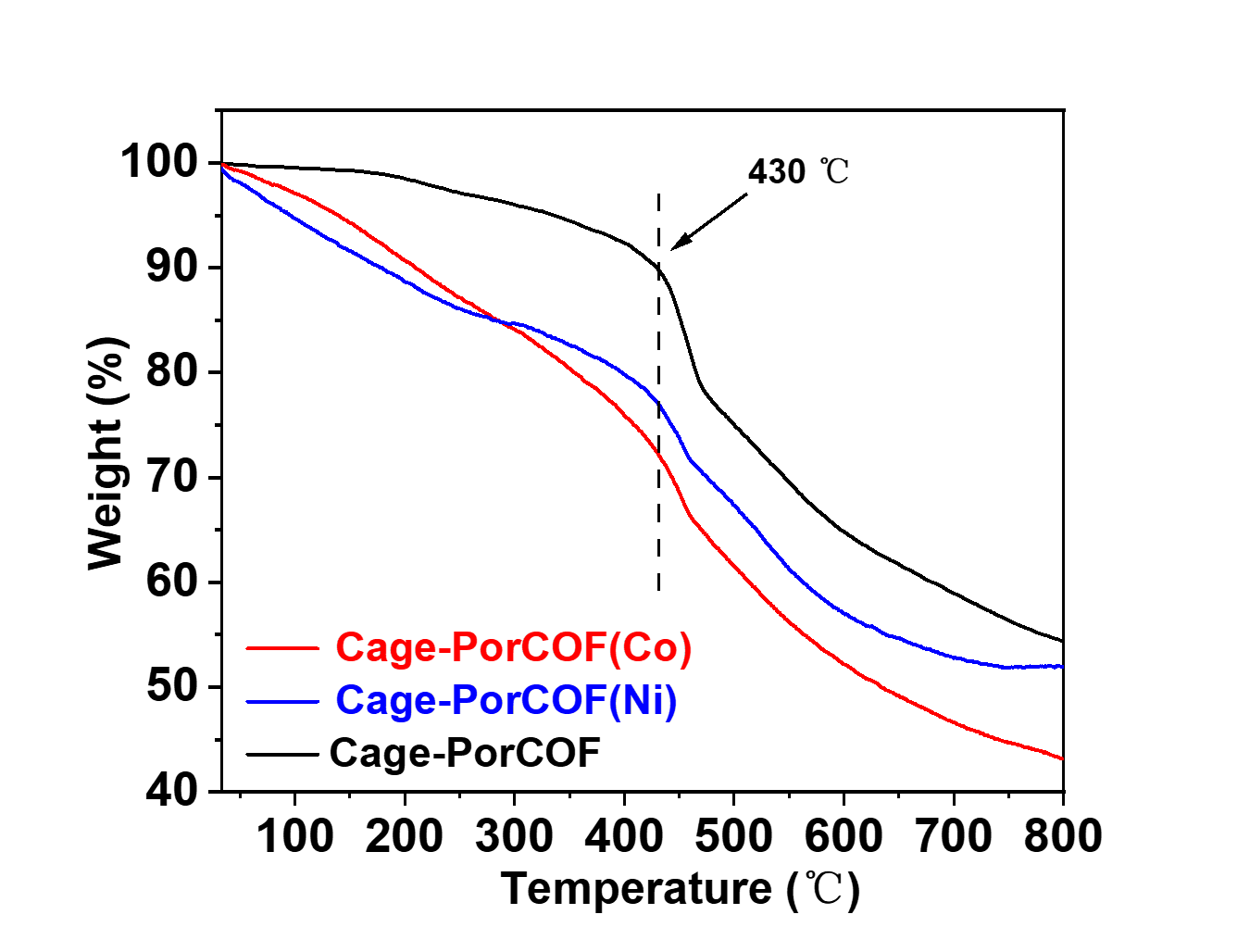


**Figure S3**. Thermogravimetric curves of **Cage-PorCOF**, **Cage-PorCOF(Co)** and **Cage-PorCOF(Ni)**.


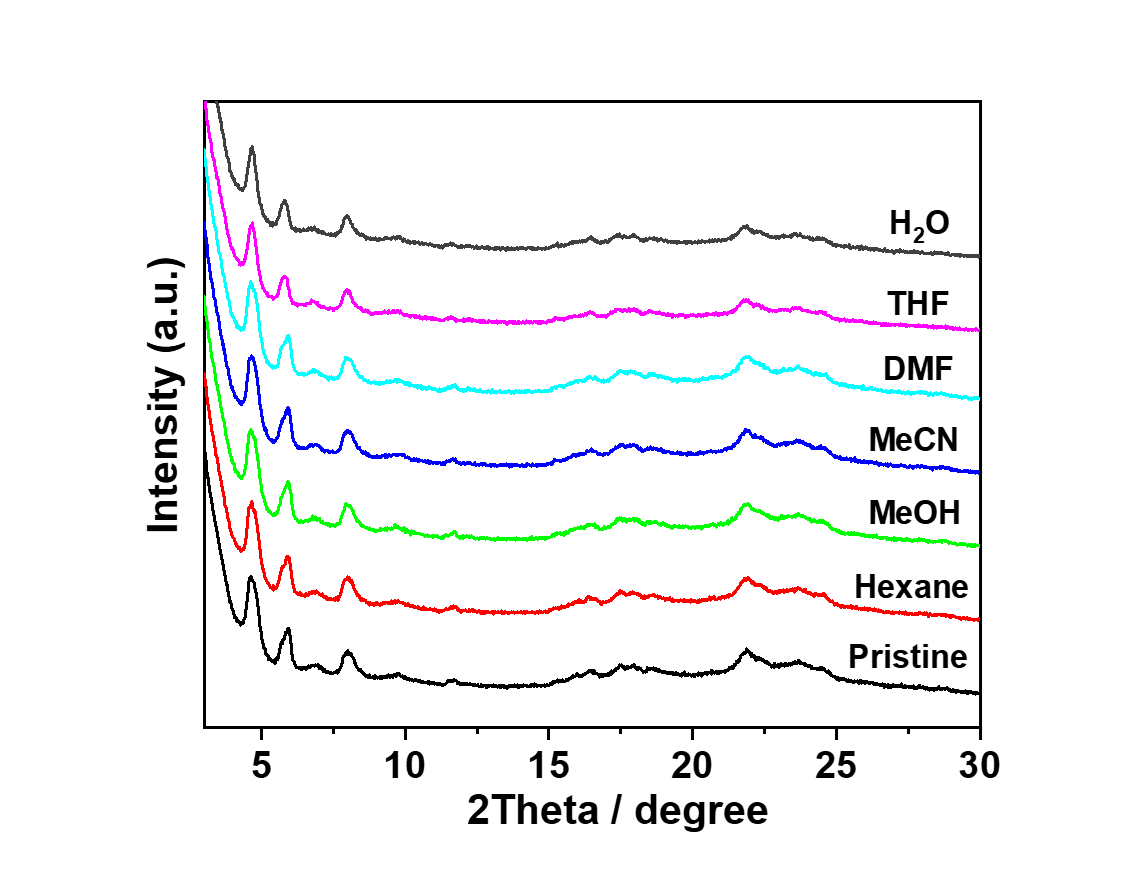


**Figure S4**. PXRD patterns of **Cage-PorCOF** after the treatment in different solvents for 24 h.


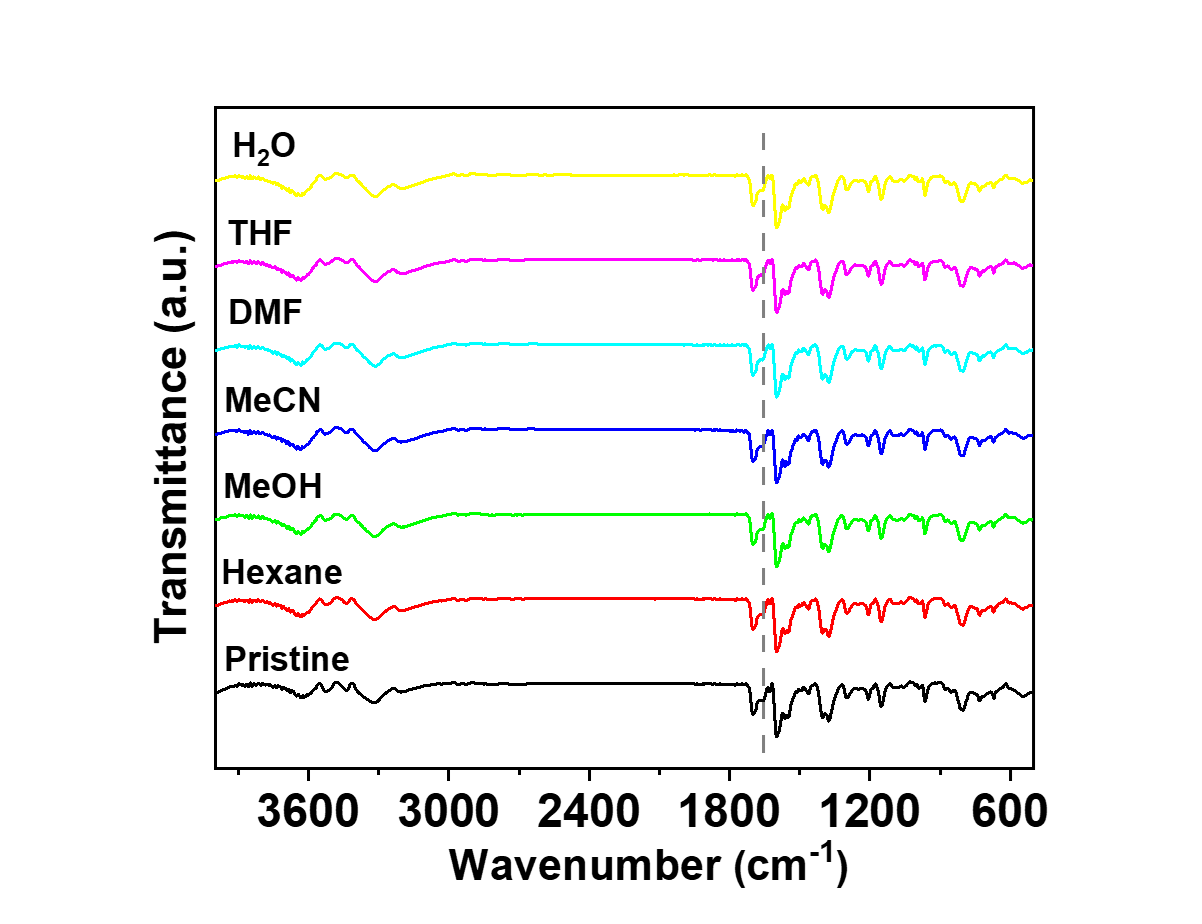


**Figure S5**. FT-IR spectra of **Cage-PorCOF** after immersed in various solvents for 24 h.


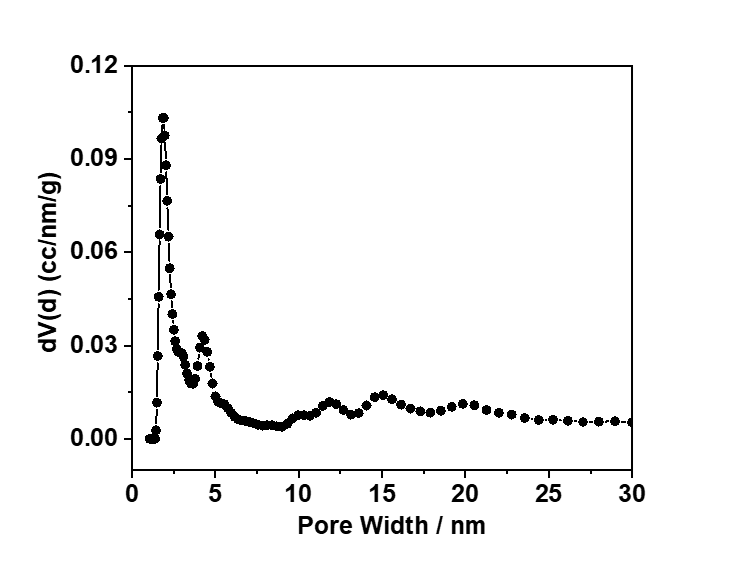


**Figure S6.** Pore size distribution of **Cage-PorCOF**.


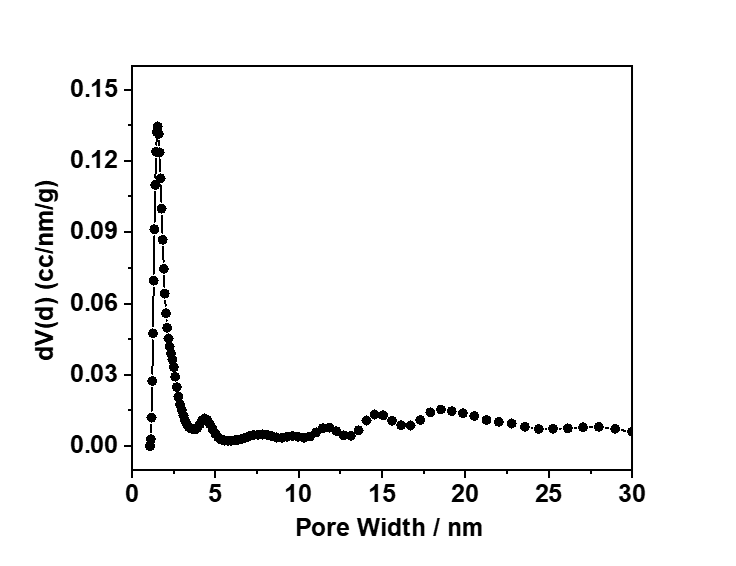


**Figure S7.** Pore size distribution of **Cage-PorCOF(Co)**.


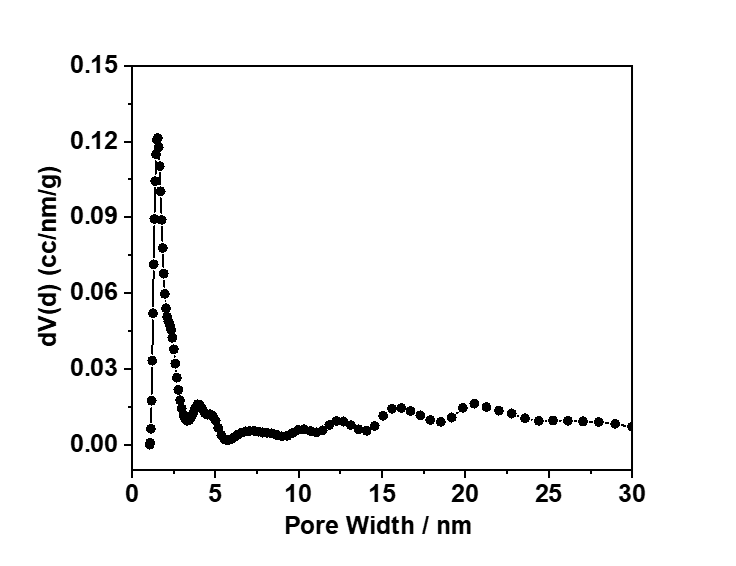


**Figure S8.** Pore size distribution of **Cage-PorCOF(Ni)**.


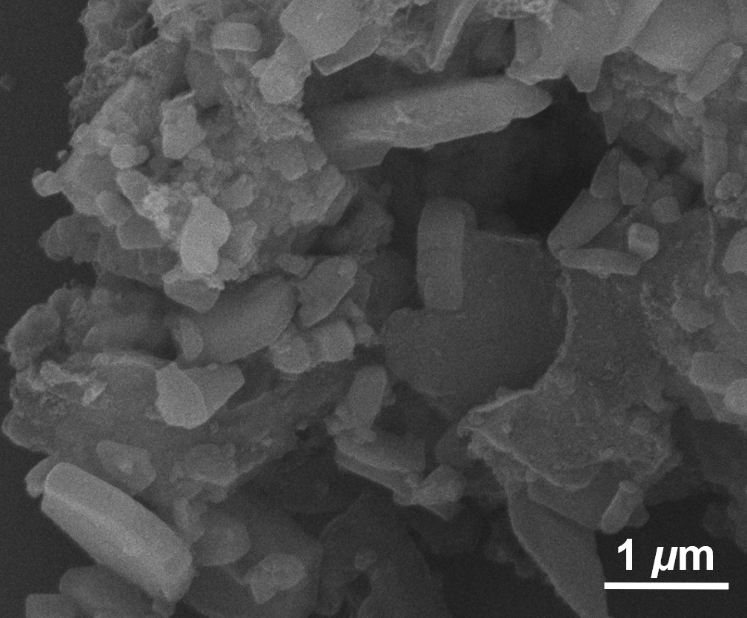


**Figure S9.** SEM image of **Cage-PorCOF.**


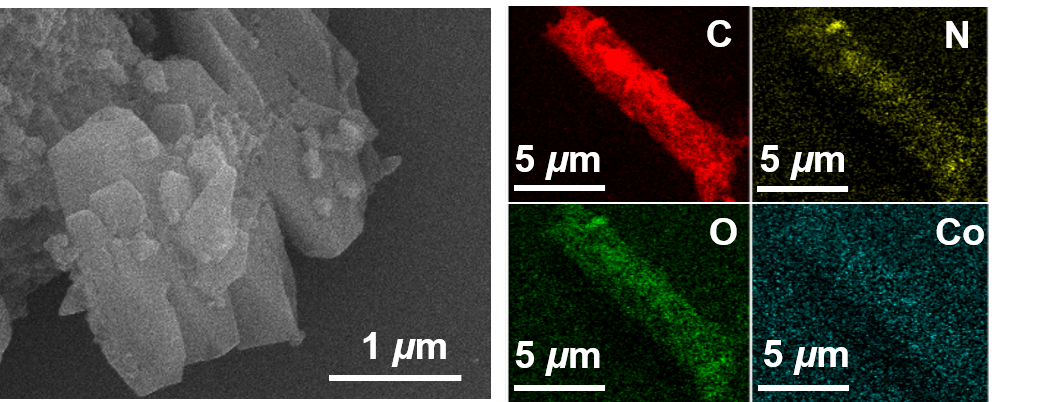


**Figure S10.** SEM image and EDS mapping images of C, O, N, and Co elements for **Cage-PorCOF(Co).**


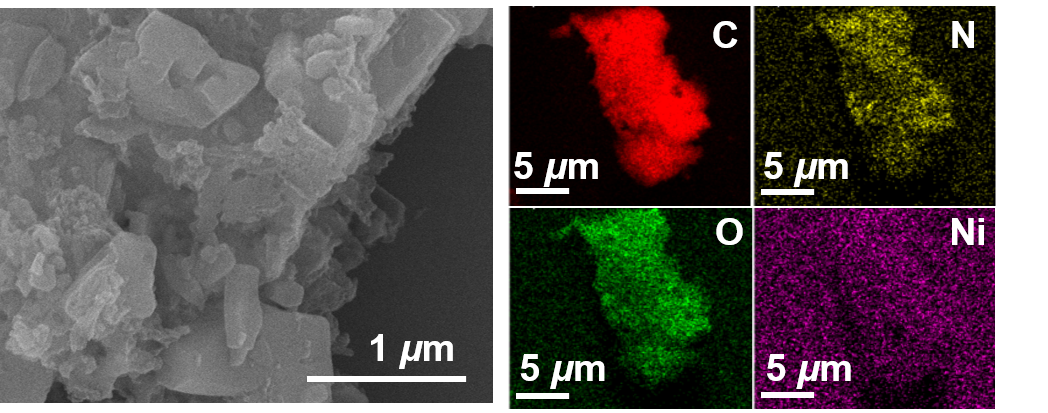


**Figure S11.** SEM image and EDS mapping images of C, O, N, and Ni elements for **Cage-PorCOF(Ni).**


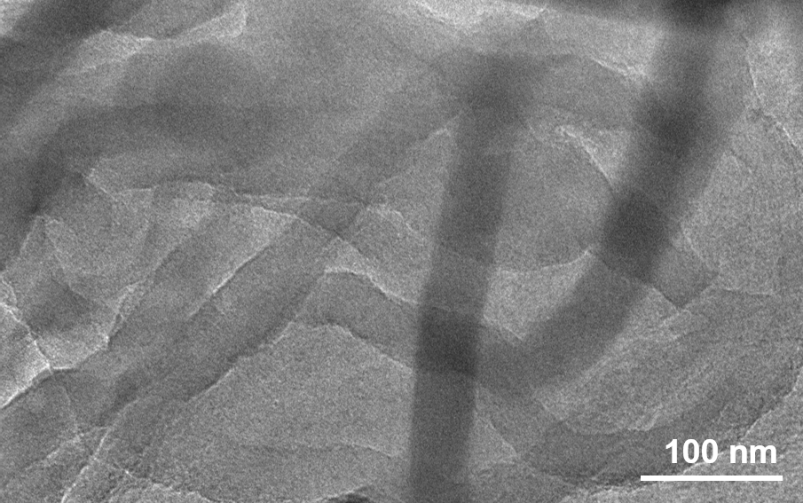


**Figure S12.** TEM image of **Cage-PorCOF(Co).**


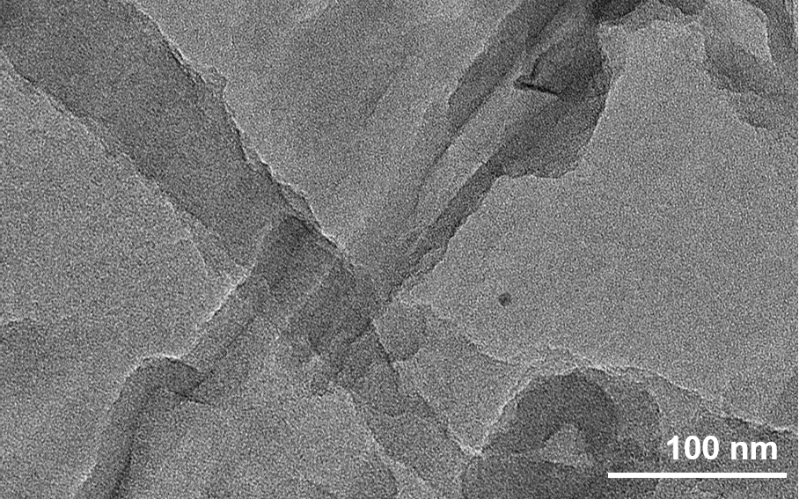


**Figure S13.** TEM image of **Cage-PorCOF(Ni).**


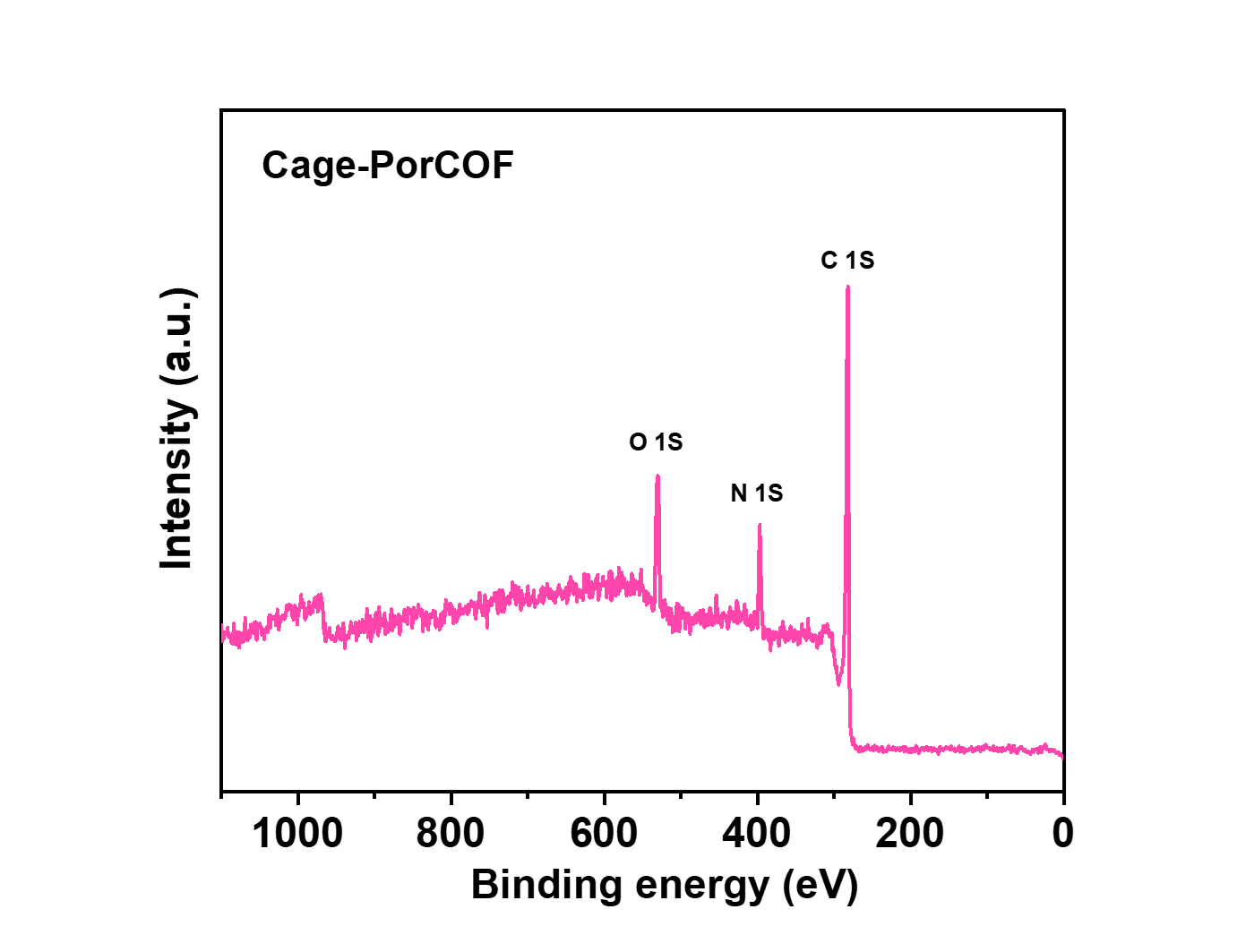


**Figure S14.** XPS broad spectrum analysis of **Cage-PorCOF**.


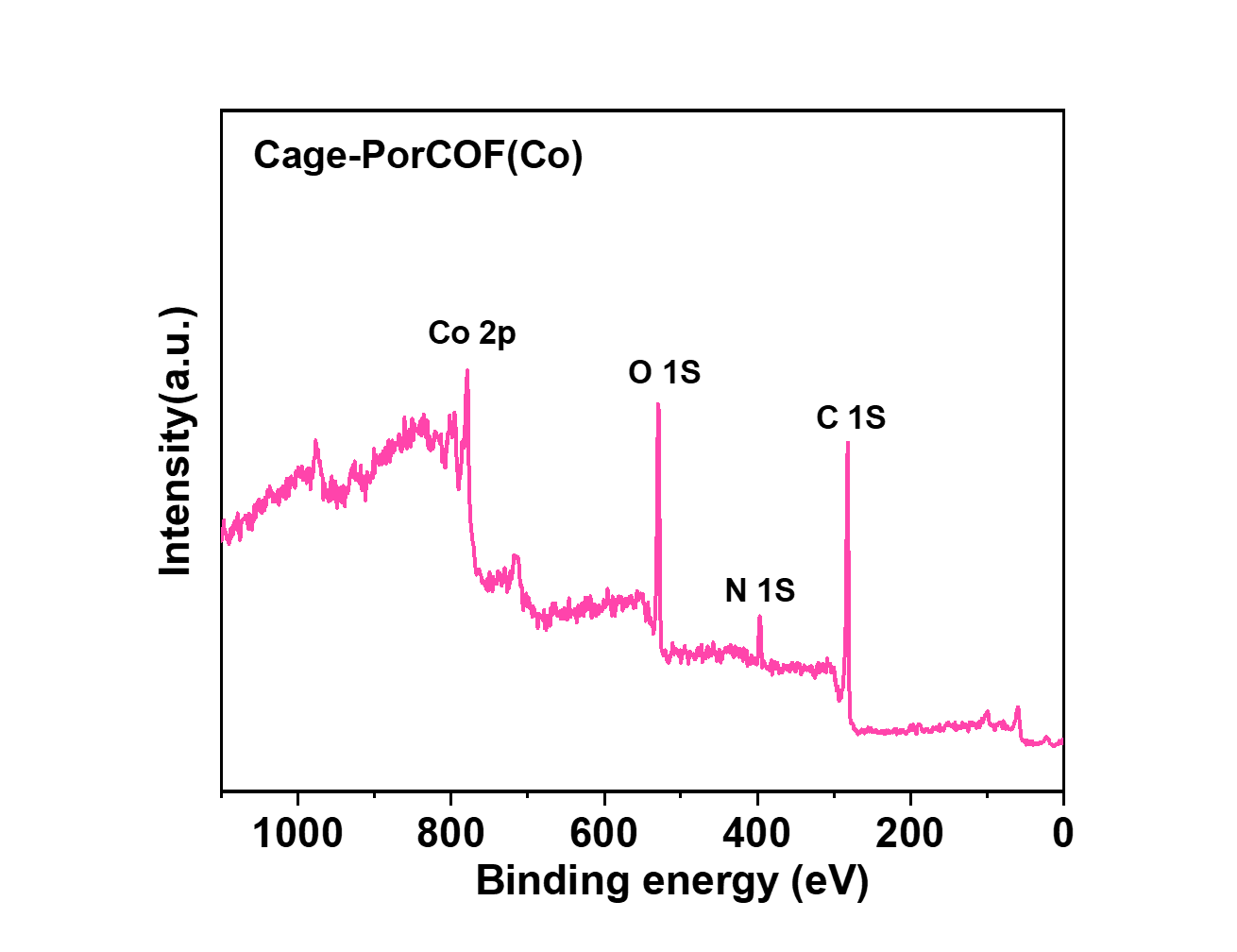


**Figure S15**. XPS broad spectrum analysis of **Cage-PorCOF(Co)**.


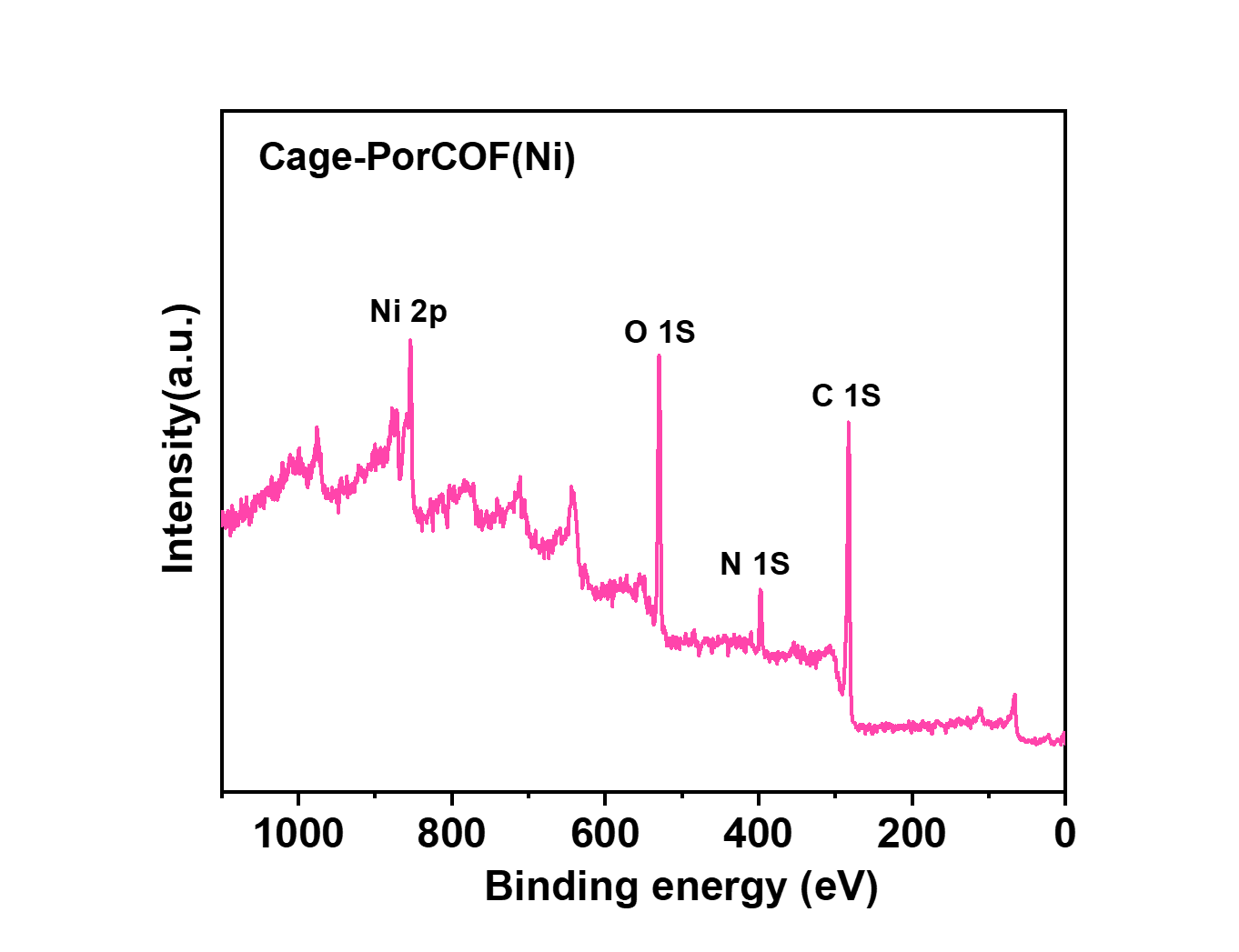


**Figure S16.** XPS broad spectrum analysis of **Cage-PorCOF(Ni)**.


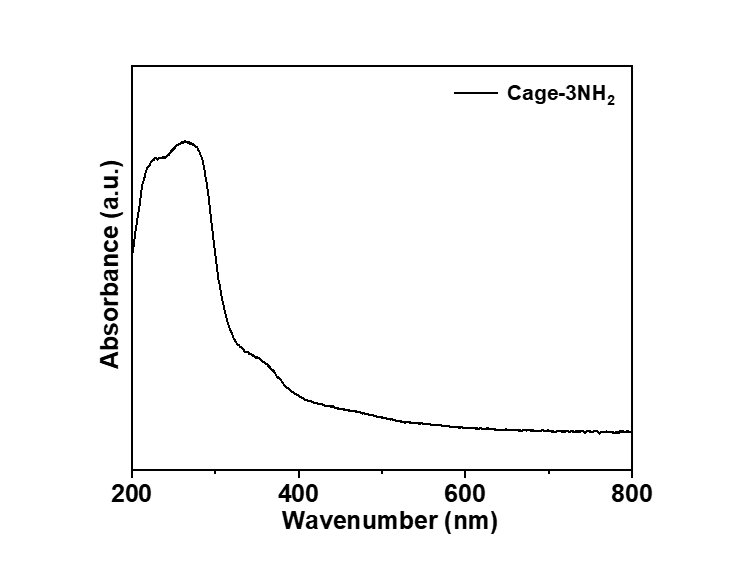


**Figure S17**. UV-vis DRS spectrum of **Cage-3NH_2_**.


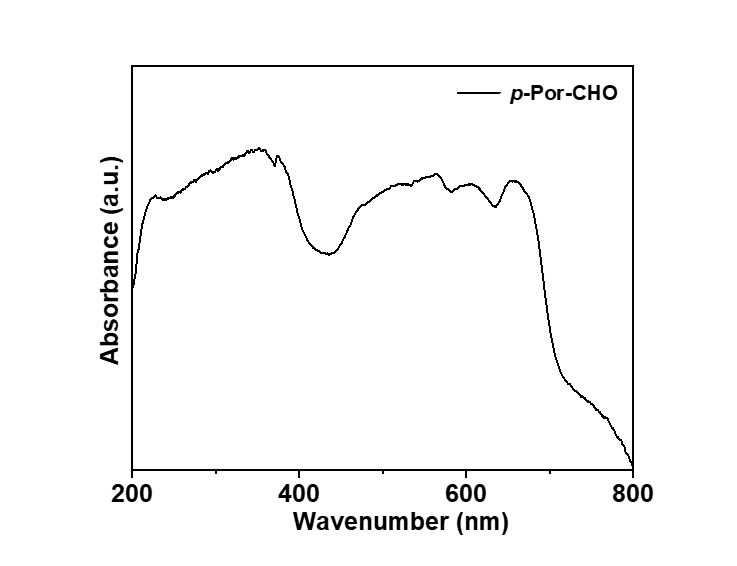


**Figure S18.** UV-vis DRS spectrum of *p*-Por-CHO.


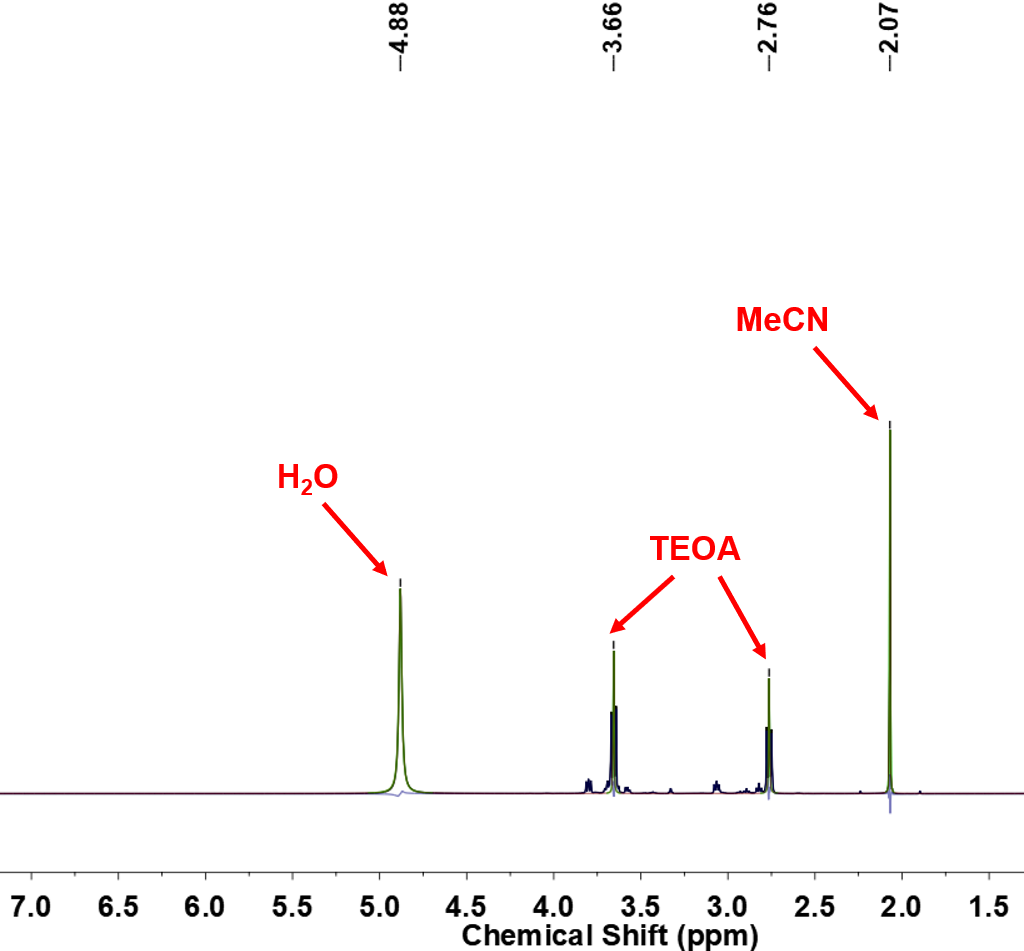


**Figure S19**. ^1^H NMR spectrum of the liquid products from the reaction system containing **Cage-PorCOF(Co)** in CD_3_OD after CO_2_ photoreduction.


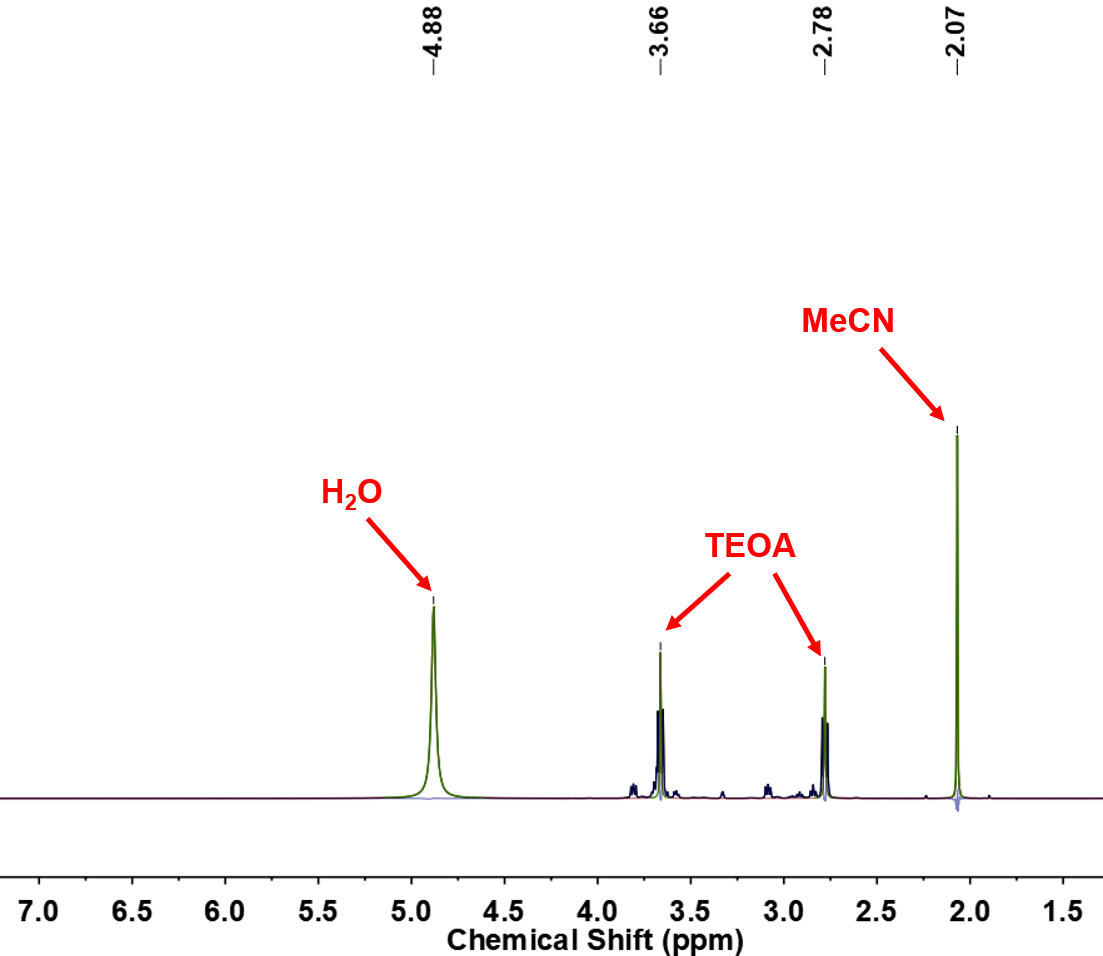


**Figure S20.** ^1^H NMR spectrum of the liquid products from the reaction system containing **Cage-PorCOF(Ni)** in CD_3_OD after CO_2_ photoreduction.


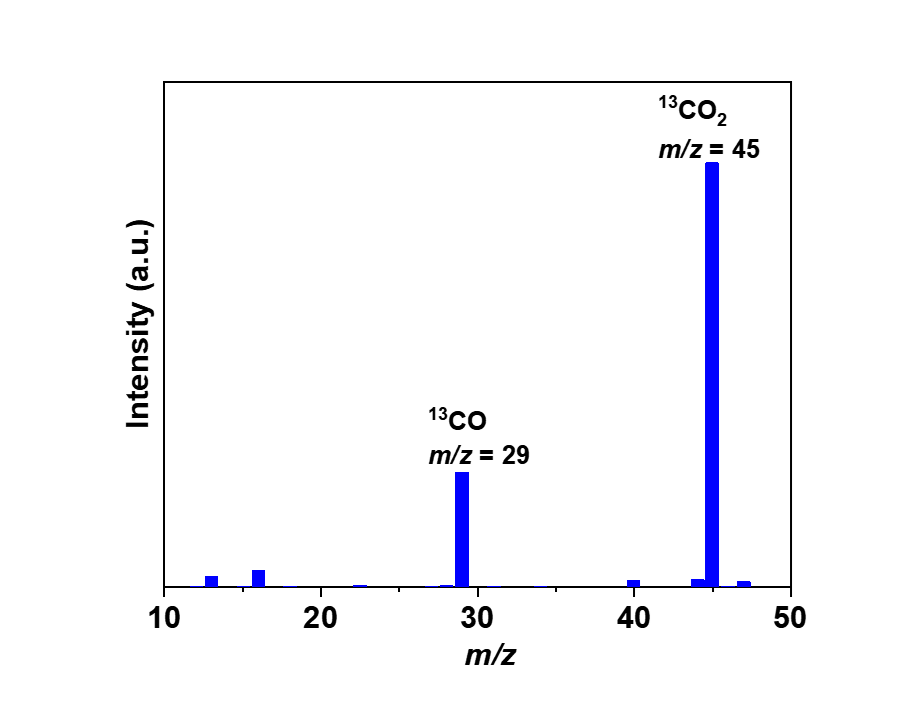


**Figure S21.** ^13^C isotope tracer results based on GC-MS for **Cage-PorCOF(Co)**.


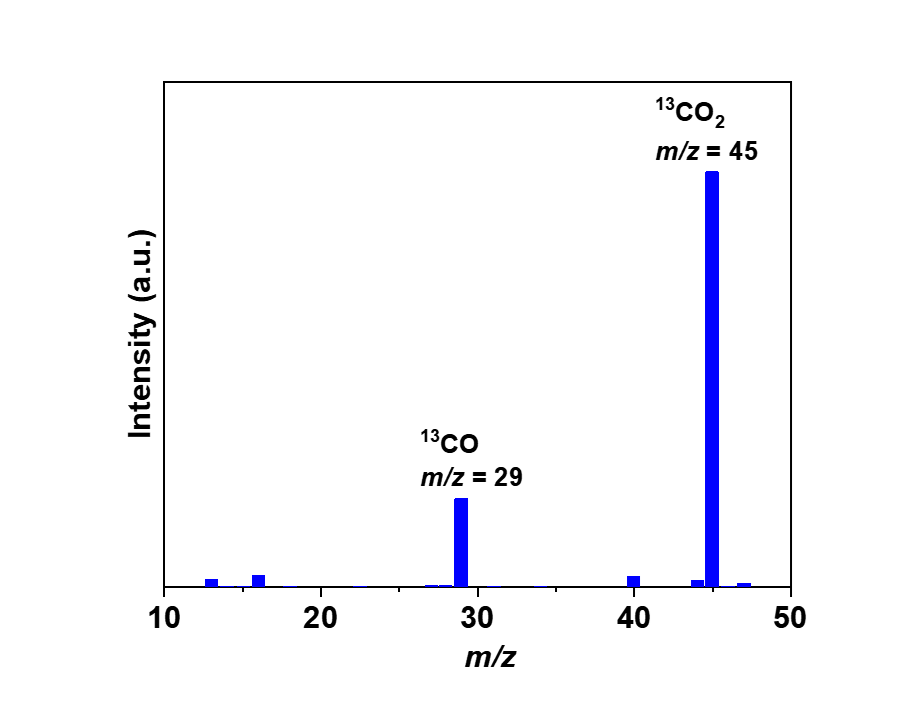


**Figure S22**. ^13^C isotope tracer results based on GC-MS for **Cage-PorCOF(Ni)**.


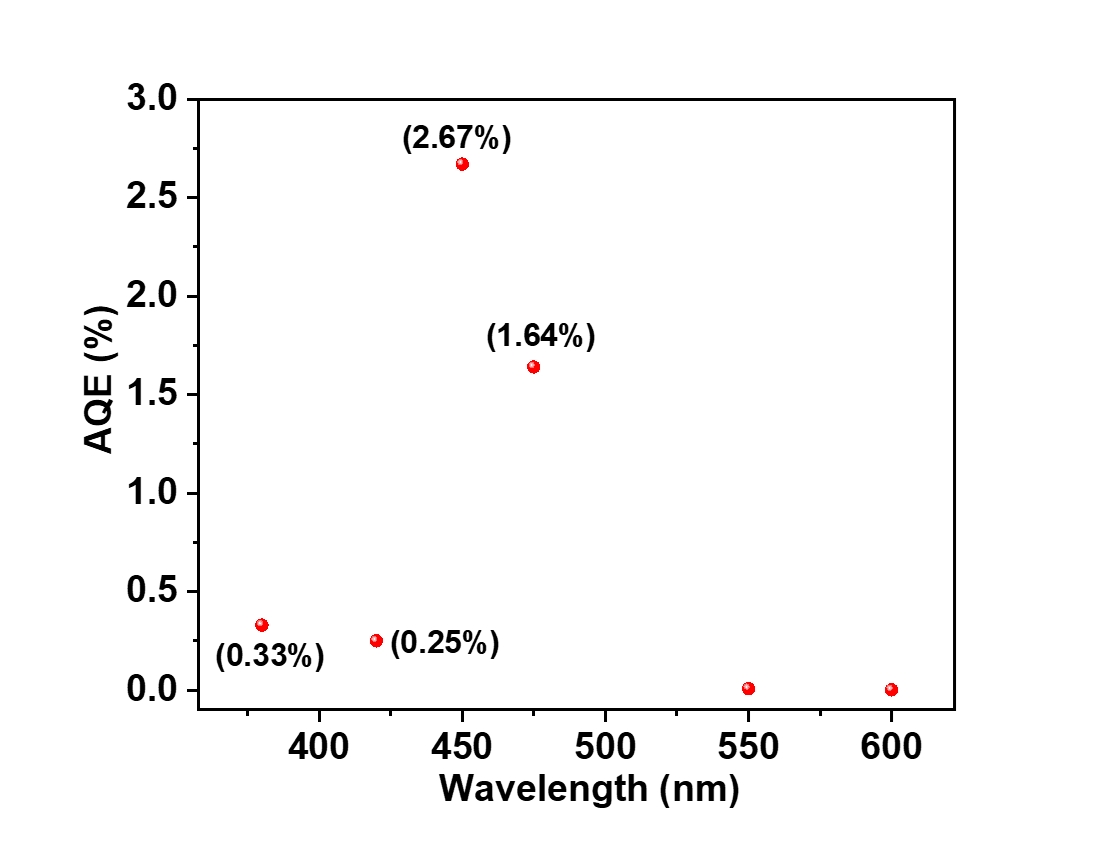


**Figure S23**. AQE for CO_2_ photoreduction of **Cage-PorCOF(Co)** at different wavelengths of monochromatic light.


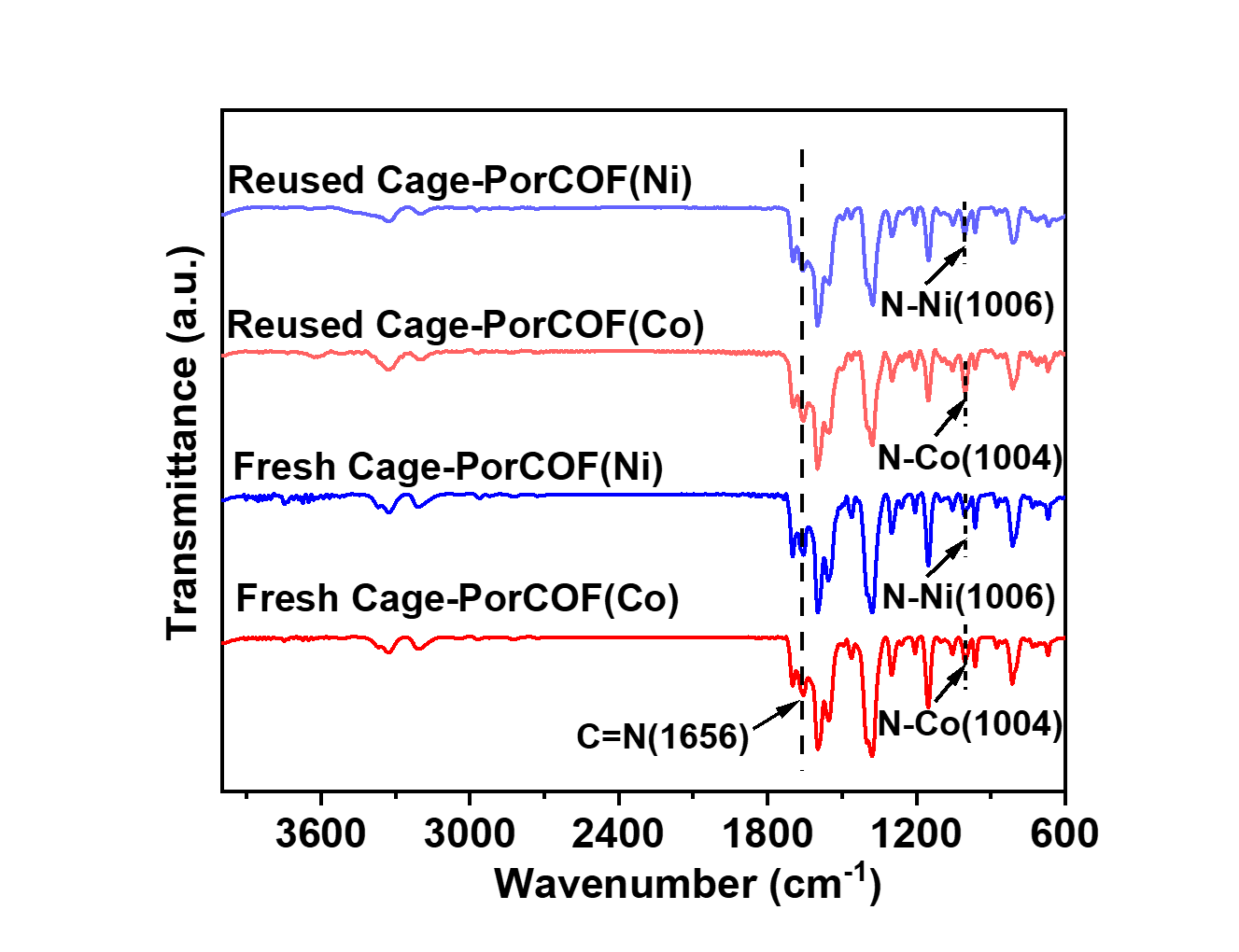


**Figure S24.** FT-IR spectra of fresh and reused **Cage-PorCOF(Co)** and **Cage-PorCOF(Ni)**.

**
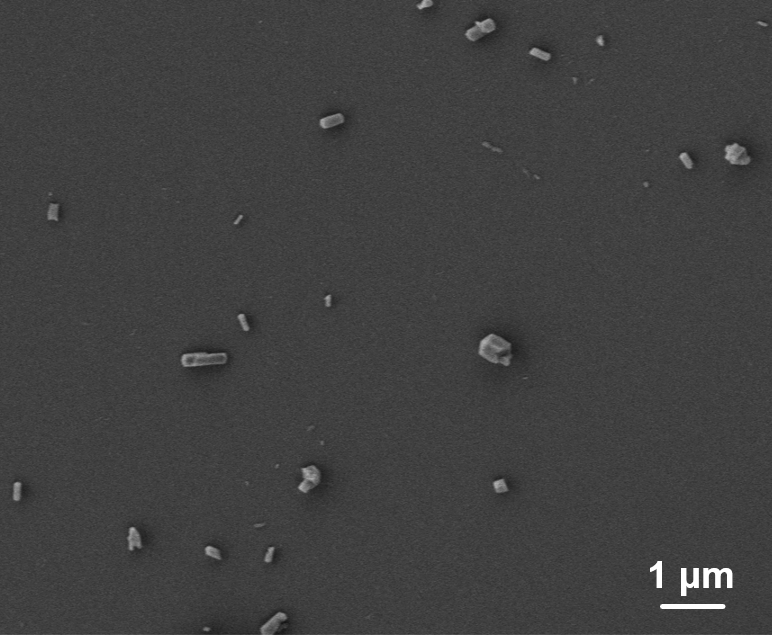
**

**Figure S25**. SEM image of **Cage-PorCOF(Co)** after photocatalysis.


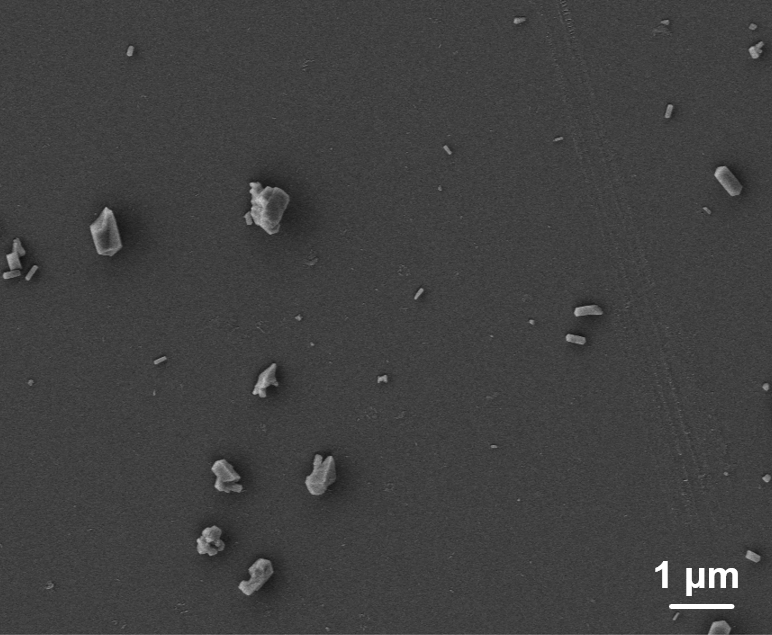


**Figure S26**. SEM image of **Cage-PorCOF(Ni)** after photocatalysis.


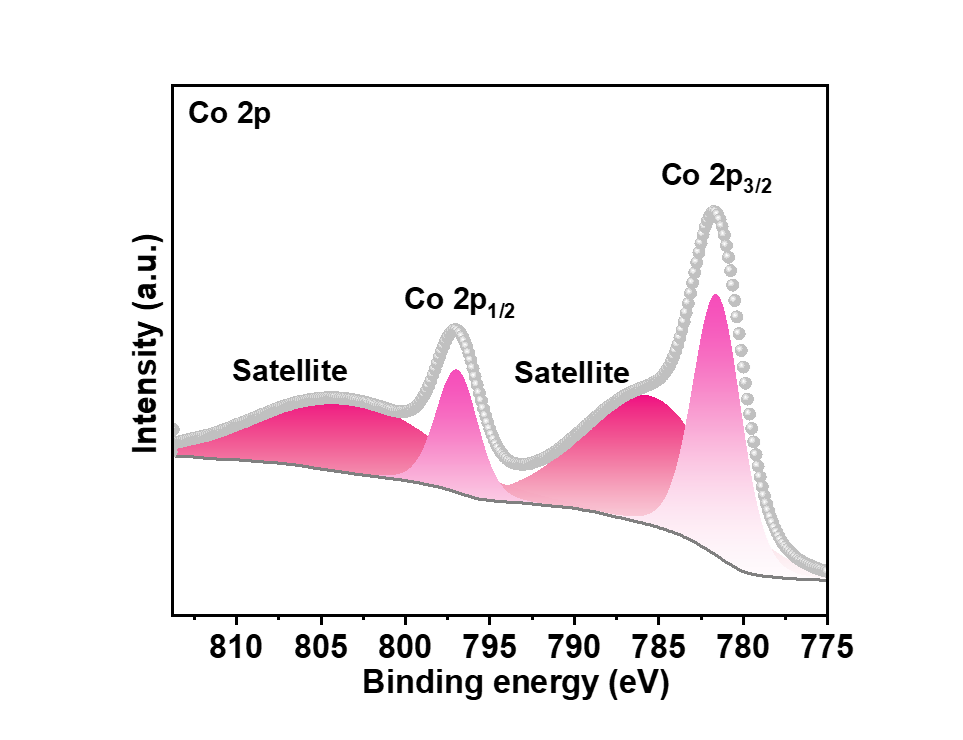


**Figure S27**. Co 2p XPS spectra of reused **Cage-PorCOF(Co)**.


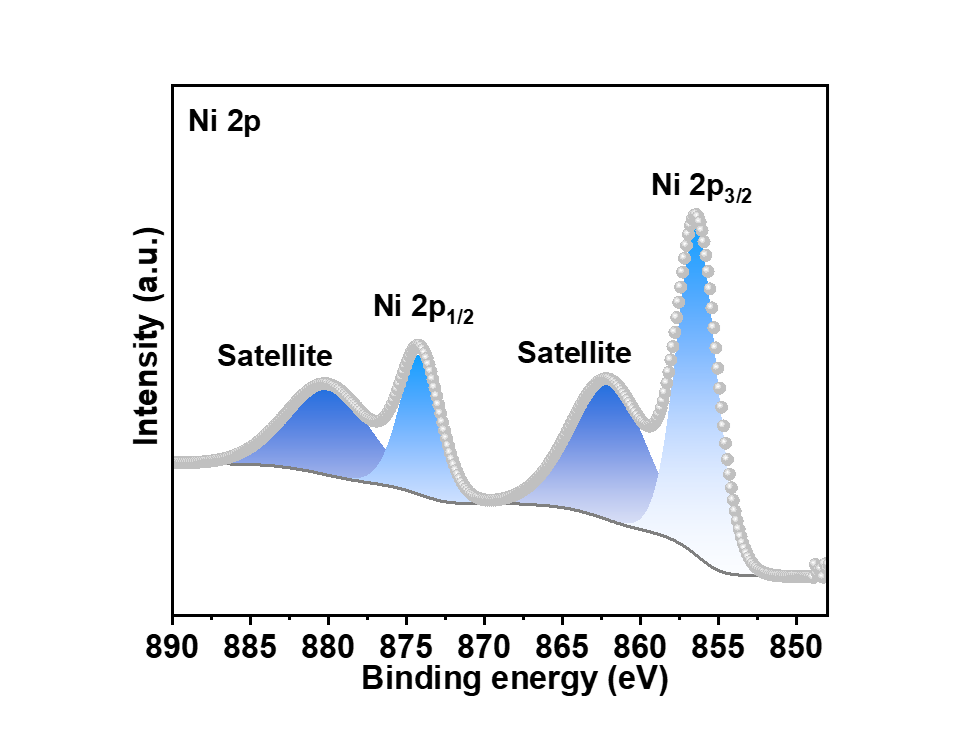


**Figure S28**. Ni 2p XPS spectra of reused **Cage-PorCOF(Ni)**.


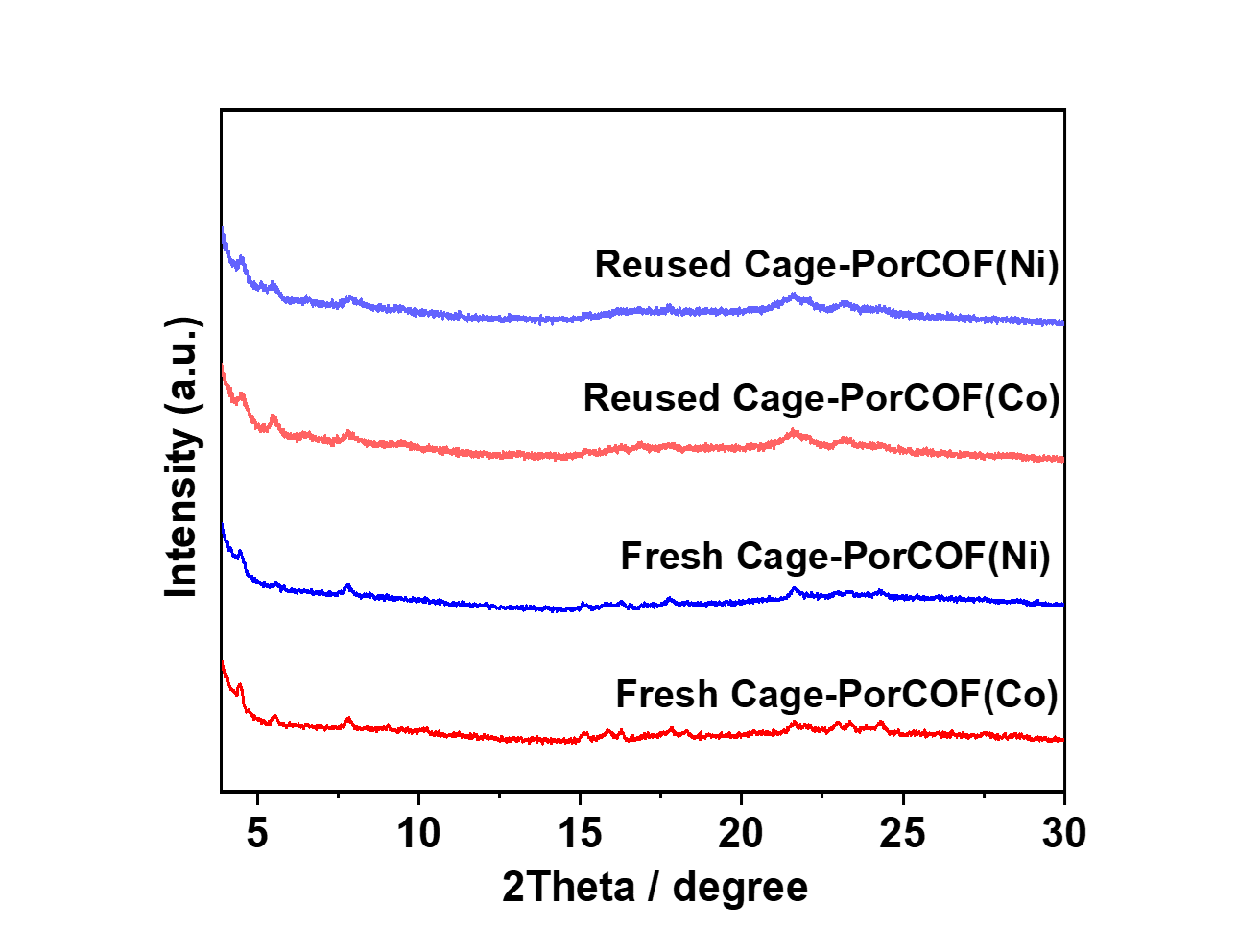


**Figure S29.** PXRD patterns of fresh and reused **Cage-PorCOF(Co)** and **Cage-PorCOF(Ni)**.


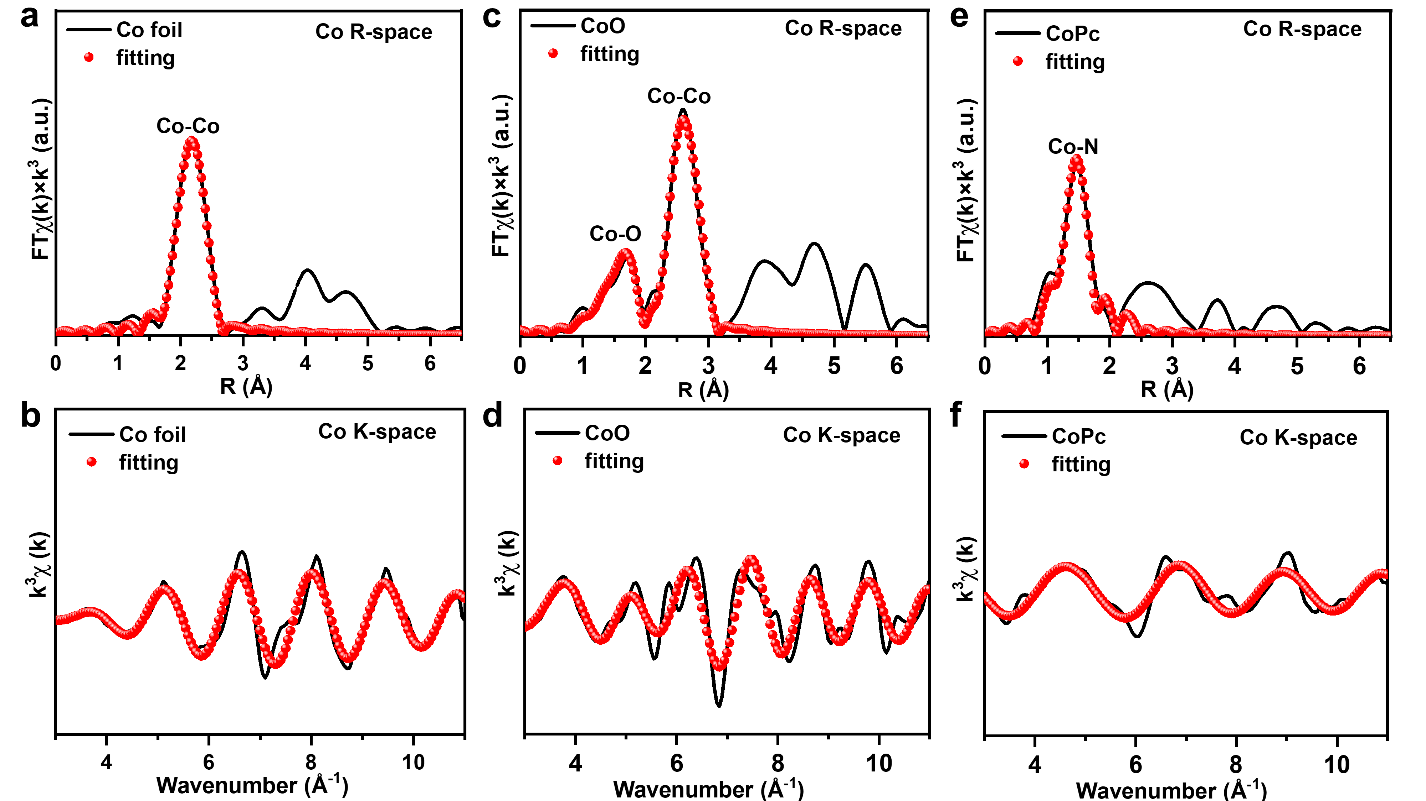


**Figure S30.** EXAFS fitting curves of (a,b) Co foil, (c,d) CoO, and (e,f) CoPc at R-space and K-space, respectively.


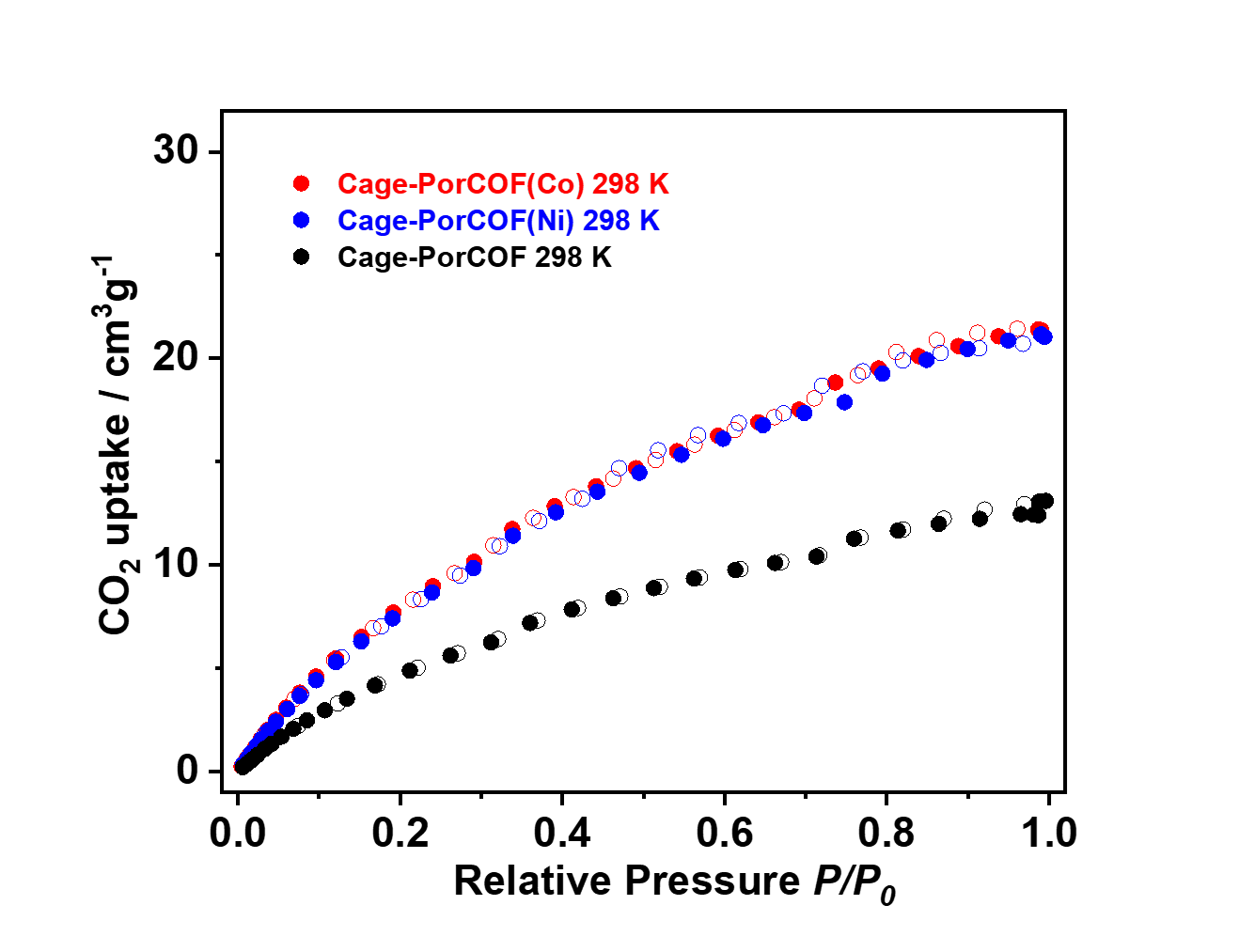


**Figure S31**. CO_2_ adsorption isotherms of **Cage-PorCOF**, **Cage-PorCOF(Co)** and **Cage-PorCOF(Ni)** at 298 K.


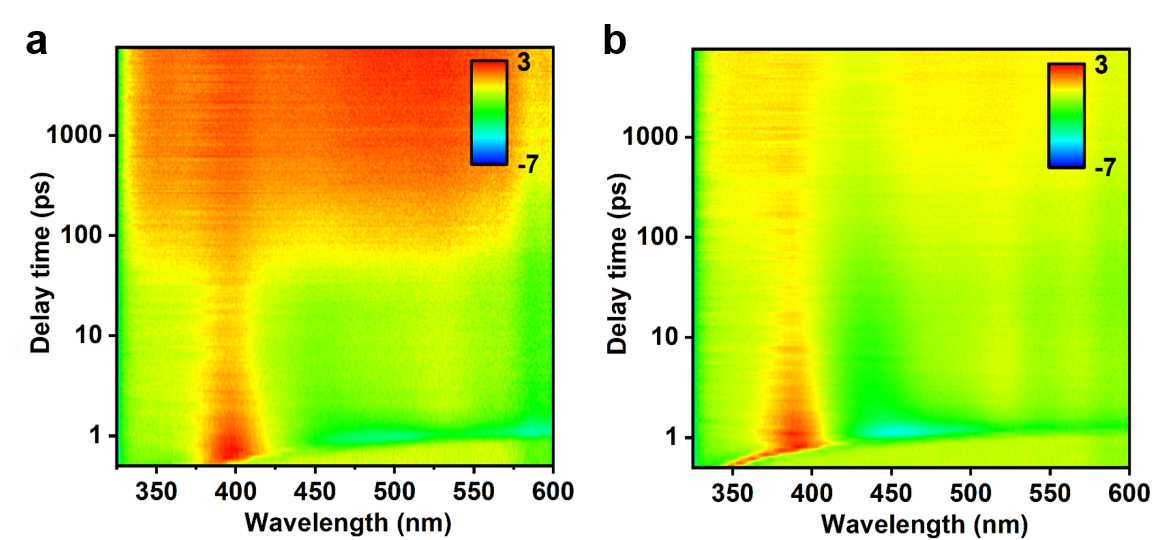


**Figure S32**. Fs-TAS of a) **Cage-PorCOF(Co)** and b) **Cage-PorCOF(Ni)**.


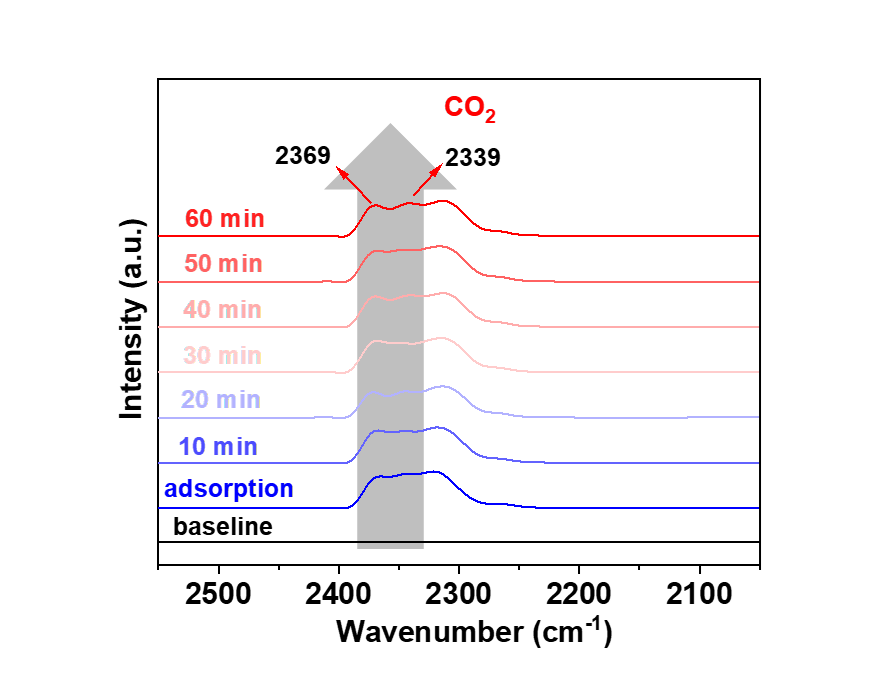


**Figure S33**. In situ DRIFTS of **Cage-PorCOF(Co)** for CO_2_ adsorption.


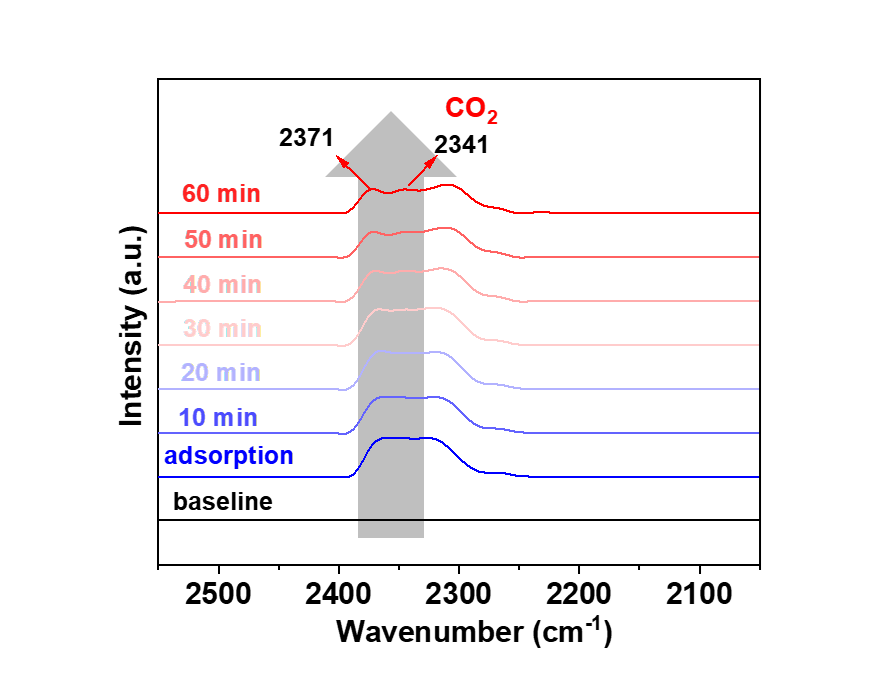


**Figure S34.** In situ DRIFTS of **Cage-PorCOF(Ni)** for CO_2_ adsorption.

**Supporting References**

[S1] D.-X. Wang, Q.-Q. Wang, Y. Han, Y. Wang, Z.-T. Huang, M.-X. Wang, *Chem. Eur. J.* **2010**, *16*, 13053-13057.

[S2] B. Han, H. Wang, C. Wang, H. Wu, W. Zhou, B. Chen, J. Jiang, *J. Am. Chem. Soc.* **2019**, *141*, 8737-8740.

[S3] Q. Zhu, J. Johal, D. E. Widdowson, Z. Pang, B. Li, C. M. Kane, V. Kurlin, G. M. Day, M. A. Little, A. I. Cooper, *J. Am. Chem. Soc.* **2022**, *144*, 9893-9901.

[S4] M. Zhang, J.-P. Liao, R.-H. Li, S.-N. Sun, M. Lu, L.-Z. Dong, P. Huang, S.-L. Li, Y.-P. Cai, Y.-Q. Lan, *Natl. Sci. Rev.* **2023**, *10*, nwad226.

[S5] Materials Studio, ver. 7.0; Accelrys Inc.; San Diego, CA, **2013**.

[S6] S. Gao, Q. Zhang, X. Su, X. Wu, X.-G. Zhang, Y. Guo, Z. Li, J. Wei, H. Wang, S. Zhang, J. Wang, *J. Am. Chem. Soc.* **2023**, *145*, 9520-9529.

[S7] G. Kresse, J. Furthmüller, *Comput. Mater. Sci.* **1996**, *6*, 15-50.

[S8] G. Kresse, J. Furthmüller, *Phys. Rev. B* **1996**, *54*, 11169-11186.

[S9] J. P. Perdew, K. Burke, M. Ernzerhof, *Phys. Rev. Lett.* **1996**, *77*, 3865-3868.

[S10] G. Kresse, D. Joubert, *Phys. Rev. B* **1999**, *59*, 1758-1775.

[S11] P. E. Blöchl, *Phys. Rev. B* **1994**, *50*, 17953-17979.

[S12] S. Grimme, J. Antony, S. Ehrlich, H. Krieg, *J. Chem. Phys.* **2010**, *132*, 154104.

[S13] S. Grimme, S. Ehrlich, L. Goerigk, *J. Comp. Chem.* **2011**, *32*, 1456-1465.

[S14] Q. Xu, J. Han, F. Tian, X. Zhao, J. Rong, J. Zhang, P. She, J.-S. Qin, H. Rao, *J. Am. Chem. Soc.* **2025**, *147*, 10587-10597.

[S15] W. Lu, C. E. Tait, G. Avci, X. e. Li, A. E. Crumpton, P. Shao, C. M. Aitchison, F. Ceugniet, Y. Yao, M. D. Frogley, D. Decarolis, N. Yao, K. E. Jelfs, I. McCulloch, *J. Am. Chem. Soc.* **2025**, *147*, 9056-9061.

[S16] D. Zhou, Q. Chen, J. Zhang, T. Wang, Z.-Q. Liu, *Angew. Chem. Int. Ed.* **2025**, e202500329.

[S17] X. Wang, X. Ding, Y. Jin, D. Qi, H. Wang, Y. Han, T. Wang, J. Jiang, *Angew. Chem. Int. Ed.* **2023**, *62*, e202302808.

[S18] Q. Zhang, S. Gao, Y. Guo, H. Wang, J. Wei, X. Su, H. Zhang, Z. Liu, J. Wang, *Nat. Commun.* **2023**, *14*, 1147.

[S19] J. Ding, X. Guan, J. Lv, X. Chen, Y. Zhang, H. Li, D. Zhang, S. Qiu, H.-L. Jiang, Q. Fang, *J. Am. Chem. Soc.* **2023**, *145*, 3248-3254.

[S20] S. Yang, W. Hu, X. Zhang, P. He, B. Pattengale, C. Liu, M. Cendejas, I. Hermans, X. Zhang, J. Zhang, J. Huang, *J. Am. Chem. Soc.* **2018**, *140*, 14614-14618.

[S21] W. Liu, X. Li, C. Wang, H. Pan, W. Liu, K. Wang, Q. Zeng, R. Wang, J. Jiang, *J. Am. Chem. Soc.* **2019**, *141*, 17431-17440.

[S22] K. H. Do, D. P. Kumar, A. P. Rangappa, J. Lee, S. Yun, T. K. Kim, *J. Mater. Chem. A* **2023**, *11*, 8392-8403.

[S23] S. Yang, R. Sa, H. Zhong, H. Lv, D. Yuan, R. Wang, *Adv. Funct. Mater.* **2022**, *32*, 2110694.

[S24] X.-Y. Dong, Y.-N. Si, Q.-Y. Wang, S. Wang, S.-Q. Zang, *Adv. Mater.* **2021**, *33*, 2101568.

[S25] W. Zhong, R. Sa, L. Li, Y. He, L. Li, J. Bi, Z. Zhuang, Y. Yu, Z. Zou, *J. Am. Chem. Soc.* **2019**, *141*, 7615-7621.

[S26] H.-L. Zheng, J.-Q. Zhao, Y.-Y. Sun, A.-A. Zhang, Y.-J. Cheng, L. He, X. Bu, J. Zhang, Q. Lin, *J. Am. Chem. Soc.* **2023**, *145*, 27728-27739.

[S27] B. Yu, L. Li, S. Liu, H. Wang, H. Liu, C. Lin, C. Liu, H. Wu, W. Zhou, X. Li, T. Wang, B. Chen, J. Jiang, *Angew. Chem. Int. Ed.* **2021**, *60*, 8983-8989.

[S28] K. Sun, Y. Huang, Q. Wang, W. Zhao, X. Zheng, J. Jiang, H.-L. Jiang, *J. Am. Chem. Soc.* **2024**, *146*, 3241-3249.

[S29] P. Dong, X. Xu, R. Luo, S. Yuan, J. Zhou, J. Lei, *J. Am. Chem. Soc.* **2023**, *145*, 15473-15481.
